# Genome-wide analysis and expression profiling of glyoxalase gene families in soybean (*Glycine max*) indicate their development and abiotic stress specific response

**DOI:** 10.1186/s12870-016-0773-9

**Published:** 2016-04-16

**Authors:** Ajit Ghosh, Tahmina Islam

**Affiliations:** Department of Biochemistry and Molecular Biology, Shahjalal University of Science and Technology, Sylhet, 3114 Bangladesh; Plant Breeding and Biotechnology Laboratory, Department of Botany, Dhaka University, Dhaka, 1000 Bangladesh

**Keywords:** Glyoxalase, *Glycine max*, Abiotic stress, Functional divergence, Gene duplication, Microarray, Metal dependency, RNA seq-Atlas, Semiquantitative RT-PCR

## Abstract

**Background:**

Glyoxalase pathway consists of two enzymes, glyoxalase I (GLYI) and glyoxalase II (GLYII) which detoxifies a highly cytotoxic metabolite methylglyoxal (MG) to its non-toxic form. MG may form advanced glycation end products with various cellular macro-molecules such as proteins, DNA and RNA; that ultimately lead to their inactivation. Role of glyoxalase enzymes has been extensively investigated in various plant species which showed their crucial role in salinity, drought and heavy metal stress tolerance. Previously genome-wide analysis of glyoxalase genes has been conducted in model plants *Arabidopsis* and rice, but no such study was performed in any legume species.

**Results:**

In the present study, a comprehensive genome database analysis of soybean was performed and identified a total of putative 41 GLYI and 23 GLYII proteins encoded by 24 and 12 genes, respectively. Detailed analysis of these identified members was conducted including their nomenclature and classification, chromosomal distribution and duplication, exon-intron organization, and protein domain(s) and motifs identification. Expression profiling of these genes has been performed in different tissues and developmental stages as well as under salinity and drought stresses using publicly available RNAseq and microarray data. The study revealed that *GmGLYI*-7 and *GmGLYII*-8 have been expressed intensively in all the developmental stages and tissues; while *GmGLYI*-6, *GmGLYI*-9, *GmGLYI*-20, *GmGLYII-*5 and *GmGLYII-*10 were highly abiotic stress responsive members.

**Conclusions:**

The present study identifies the largest family of glyoxalase proteins to date with 41 GmGLYI and 23 GmGLYII members in soybean. Detailed analysis of *GmGLYI* and *GmGLYII* genes strongly indicates the genome-wide segmental and tandem duplication of the glyoxalase members. Moreover, this study provides a strong basis about the biological role and function of GmGLYI and GmGLYII members in soybean growth, development and stress physiology.

**Electronic supplementary material:**

The online version of this article (doi:10.1186/s12870-016-0773-9) contains supplementary material, which is available to authorized users.

## Background

The glyoxalase system is a two-enzyme driven pathway that detoxifies the highly cytotoxic compound, methylglyoxal (MG) to D-lactate. The detoxification is accomplished by the sequential action of two thiol-dependent enzymes; glyoxalase І (GLYI) and glyoxalase II (GLYII). In presence of reduced glutathione (GSH), MG is converted into hemithioacetal (HTA) spontaneously, and GLYI catalyses the isomerization of this HTA into S-D-lactoyl-glutathione (SLG). GLYII hydrolyses SLG into D-lactate and recycles back one molecule of GSH to the system [1]. Both, the formation of MG and the glyoxalase enzymes have been ubiquitously found in all organisms from *Escherichia coli* to *Homo sapiens* [2].

Besides its proposed role in the detoxification of MG as metabolic enzyme, glyoxalase enzymes have been reported to be involved in various other functions. Glyoxalase system protects human from various vascular complications of diabetes, such as nephropathy, retinopathy, neuropathy and cardiovascular disease by resisting the increased accumulation of MG [3]. Moreover, glyoxalase pathway has also been shown to be involved in different important cellular functions of human, such as cell division and proliferation, microtubule assembly and protection against oxoaldehydes toxicity [4]. For this, the pathway has been regarded as “marker for cell growth and division”. Similarly, stress tolerance potential of glyoxalase has been reported in plant by numerous studies [5]. Transgenic plants over-expressing GLYI and/or GLYII were found to provide significant tolerance against multiple abiotic stresses including salinity, drought and heavy metal toxicity [5, 6]. Thus MG and glyoxalases are considered as potential biomarkers for plant stress tolerance [7].

Glyoxalase proteins have been extensively characterized from different genera such as *Escherichia coli*, *Homo sapiens*, *Saccharomyces cerevisiae*, *Arabidopsis thaliana* and *Oryza sativa* [2]. Compared to other organisms, very little is known about plant glyoxalases. The first plant glyoxalase activity was reported from Douglas fir needles by Smits and Johnson [5]. Thereafter, presence of glyoxalase activity has been reported from various other plant species, such as rice, *Arabidopsis*, tomato, wheat, sugarcane, *Brassica* etc. [7]. Most of the genes of plant exist as family due to the expansion and gene duplication during the course of plant evolution [8]. Availability of the whole genome sequences has opened up the field to identify and characterize plant glyoxalase family substantially. According to *in silico* genome wide analyses of rice and *Arabidopsis*, there are eleven potential *GLYI* and three *GLYII* genes in rice; and eleven *GLYI* and five *GLYII* genes in *Arabidopsis* [1]. Expression analysis of all these genes have been performed in different developmental tissues and stages, and in response to multiple abiotic stresses using publicly available MPSS and microarray database. It has been observed that *AtGLYI-3*, *OsGLYI-11, AtGLYII-2*, *AtGLYII-5*, *OsGLYII-2* and *OsGLYII-3* showed constitutive expression in all the tissues and stages, while *AtGLYI-8*, *OsGLYI-3*, and *OsGLYI-10* expressed only in seed [1]. On the other hand, *AtGLYI-7*, *OsGLYI-11*, *AtGLYII-2* and *OsGLYII-3* were the most stress inducible members [1].

Among these identified glyoxalase members, *GLYII* genes have been extensively studied from both rice and *Arabidopsis* but the research on *GLYI* is still very limited. To date, all five *AtGLYII* and three *OsGLYII* genes have been well characterized. Both *OsGLYII-2* and *OsGLYII-3* possessed typical GLYII enzymatic activity and overexpression of these genes in tobacco provides enhanced tolerance against salinity stress [9, 10]. However, OsGLYII-1, along with AtGLYII-5 showed functional divergence by possessing sulphur dioxygenase (SDO) activity instead of GLYII [11]. One of the rice GLYI, OsGLYI-11.2 have been studied extensively and found to possess Ni^2+^-dependent GLYI activity with stress modulation potential [12].

Soybean (*Glycine max* [L.] Merr.) is a legume plant of Papilionoideae family [13], major source of vegetable protein and edible oil. It also has the capacity to fix atmospheric nitrogen through symbioses [14]. However, production of soybean is under threat due to the unfavourable environmental stimuli such as drought, salinity and osmotic stresses [15, 16]. All these stresses severely affect the overall plant development in all the stages from germination to flowering and reduce the productivity and seed quality of soybean. The yield has been reported to be reduced by about 40 % in response to drought [15]. Thus, there is an urgent need to identify novel stress responsive soybean genes using the available genome database [14]. The soybean genome contains 46,430 predicted protein-coding genes which are 70 % more than *Arabidopsis*. There have been two genome duplication events undergone in soybean at approximately 59 and 13 million years ago, that resulted a highly duplicated genome (more than 75 % of the genes are duplicated) [14]. A lot of gene families have been studied in soybean, such as ERF, HD-Zip, WRKY, BURP, MADS-box, MYB, NAC, CYP [13, 17–22].

Genome wide analyses of glyoxalase gene family have been done in *Arabidopsis* and rice [1], but no such analysis has been performed in soybean in spite of having a handful genome sequences deposited in the publicly available database. Here, we present a detailed genome-wide identification of soybean *GLYI* and *GLYII* genes, their phylogenetic relationship, chromosomal distribution, structural and expressional analysis. Present results indicate that soybean genome contains 41 GLYI and 23 GLYII proteins, the largest family of glyoxalase known to date in any organism. Expression analysis of these genes based on publicly available microarray data indicates the differentially regulation of glyoxalase members in response to various developmental cues as well as stress treatments. In particular *GmGLYI*-6, *GmGLYI*-9 and *GmGLYII*-5 are most up-regulated stress responsive members that might resist MG accumulation in stress by interacting with other members. This study will facilitate the further investigation of soybean glyoxalase genes for the biological and molecular functions.

## Results

### Identification of *GLYI* and *GLYII* gene families in soybean

**Fig. 1:**
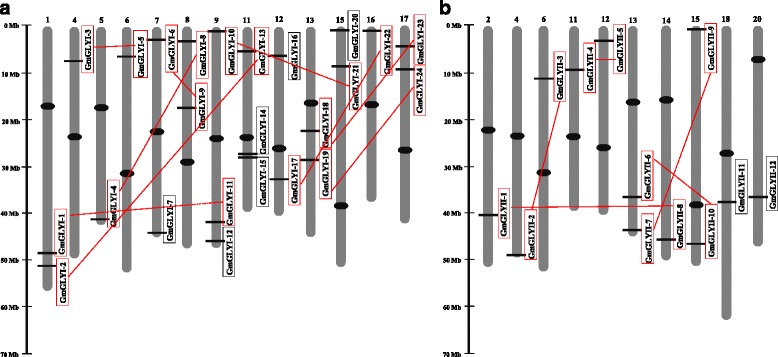
Chromosomal distribution of *GmGLYI* (**a**) and *GmGLYII* (**b**) genes on different soybean chromosomes. Only the chromosomes having glyoxalase genes are shown and their number is indicated above by Roman numbers. The scale is in mega base (Mb), and the centromeric regions are indicated by black ellipses. Red coloured boxes indicate the segmental duplicated genes connected by red lines, based on sequence similarities and divergence analysis (Table 3). Black boxes indicate the non-duplicated genes

Proteins having lactoylglutathione lyase domain (PF00903) have been classified as GLYI proteins and metallo-beta-lactamase domain (PF00753) have been classified as GLYII proteins [1]. Previously, glyoxalase proteins have been identified in two model plant genome, *Arabidopsis* and rice [1]. To identify all the putative members of the glyoxalase proteins in soybean, a BLASTP search of the soybean genome database *G. max* Wm82.a2.v1 (http://phytozome.jgi.doe.gov/pz/portal.html#!search?show=BLAST&method=Org_Gmax) was performed using the previously characterized protein sequence as a query. GLYI proteins have been primarily identified using a previously reported soybean GLYI protein (GenBank: NM_001249223.1). Subsequently, each of the newly identified GLYI protein sequences has been used as a query sequence individually in BLASTP search of soybean genome database. Subsequent searching process was repeated until there was no new member documented. This search resulted in the identification of total 43 unique proteins. All these identified proteins were analyzed using Pfam to check the presence of unique lactoylglutathione lyase domain (PF00903). This analysis discarded two members due to the lack of lactoylglutathione lyase domain, and finally landed to a total of 41 soybean GLYI proteins which is greater than the previously reported *Arabidopsis* (22) and rice (19) GLYI proteins. These 41 GLYI proteins have been coded by 24 unique genes located on 13 different chromosomes (Fig. 1). They were identified and named as *GmGLYI*-1 to *GmGLYI*-24 following the nomenclature proposed previously [1] (Table 1).

**Table 1:** List of identified *GLYI* genes in Soybean (*Glycine max*) along with their detailed information and localization

Name	Gene	Protein	Chro. no	CDS coordinate (5’ to 3’)	CDS (bp)	Exons	PP length (aa)	MW (kDa)	pI	Localization
GmGLYI-1	Glyma.01 g146300	Glyma.01 g146300.1	1	48150827–48154366	1044	9	347	39.3	7.01	Ch^a,c^; Mt^b^; Cy^b^
		Glyma.01 g146300.2			1038	9	345	39.1	7.01	Ch^a,c^; Mt^b^; Cy^b^
		Glyma.01 g146300.3			930	8	309	35.1	8.44	Ch^a,b,c^; Mt^b^; Cy^b^
GmGLYI-2	Glyma.01 g168400	Glyma.01 g168400.1	1	50605679–50608020	579	3	192	22.0	5.04	Cy^a^; Ec^b^
GmGLYI-3	Glyma.04 g083100	Glyma.04 g083100.1	4	7006088–7009361	1041	9	346	38.5	5.83	Ch^a,b,c^
GmGLYI-4	Glyma.05 g228500	Glyma.05 g228500.1	5	40646576–40651812	1101	9	366	40.6	8.17	Ch^a,b,c^
		Glyma.05 g228500.2			1089	9	362	40.2	6.28	Ch^a,b,c^
GmGLYI-5	Glyma.06 g084500	Glyma.06 g084500.1	6	6498718–6501298	792	8	263	29.7	5.45	Cy^b^
GmGLYI-6	Glyma.07 g031700	Glyma.07 g031700.1	7	2508024–2510223	519	2	172	19.6	5.10	Nu^a^; Cy^b^
		Glyma.07 g031700.2			369	3	122	13.9	4.89	Cy^a,b^
		Glyma.07 g031700.3			381	2	126	14.5	5.03	Cy^a,b^
GmGLYI-7	Glyma.07 g261400	Glyma.07 g261400.1	7	43645750–43649851	843	7	280	31.6	5.62	Cy^a,b^
		Glyma.07 g261400.2			732	8	243	27.2	5.34	Cy^a,b^
		Glyma.07 g261400.3			795	7	264	29.8	5.24	Cy^a,b^
		Glyma.07 g261400.4			843	8	280	31.6	5.62	Cy^a,b^
		Glyma.07 g261400.5			843	8	280	31.6	5.62	Cy^a,b^
		Glyma.07 g261400.6			819	8	272	30.7	6.11	Cy^a,b^
GmGLYI-8	Glyma.08 g035400	Glyma.08 g035400.1	8	2811954–2818455	1071	9	356	39.6	6.56	Ch^a,b,c^
GmGLYI-9	Glyma.08 g211100	Glyma.08 g211100.1	8	17046316–17048640	426	3	141	16.4	4.86	Ch^a^;Nu^a^; Cy^a,b^
GmGLYI-10	Glyma.09 g004300	Glyma.09 g004300.1	9	340275–344376	975	8	324	36.9	6.62	Mt^a^; Cy^b^
		Glyma.09 g004300.2			891	9	296	33.5	6.13	Cy^a,b^
		Glyma.09 g004300.3			864	9	287	32.4	5.74	Cy^a,b^
		Glyma.09 g004300.4			840	8	279	31.5	6.97	Cy^a,b^
		Glyma.09 g004300.5			951	9	316	35.9	8.45	Mt^a,b^; Cy^b^
GmGLYI-11	Glyma.09 g193800	Glyma.09 g193800.1	9	41830881–41834537	1041	9	346	39.0	6.68	Ch^a,b,c^; Mt^b^
		Glyma.09 g193800.2			819	7	272	30.6	8.76	Ch^a,c^; Mt^b^
GmGLYI-12	Glyma.09 g226500	Glyma.09 g226500.1	9	45136253–45137166	333	2	110	12.8	5.23	Cy^b^
GmGLYI-13	Glyma.11 g075000	Glyma.11 g075000.1	11	5598647–5600229	579	3	192	22.1	4.97	Cy^a,b^
GmGLYI-14	Glyma.11 g194200	Glyma.11 g194200.1	11	26776807–26779603	747	8	248	28.5	5.48	Po^a^; Cy^b^; Ec^b^
GmGLYI-15	Glyma.11 g194300	Glyma.11 g194300.1	11	26780838–26784795	702	8	233	26.5	9.16	Ch^a,c^; Mt^b^
GmGLYI-16	Glyma.12 g079700	Glyma.12 g079700.1	12	6241940–6246776	708	7	235	26.8	9.28	Mt^a,b^
		Glyma.12 g079700.2			558	6	185	21.0	5.41	Cy^a,b^
		Glyma.12 g079700.3			525	8	174	20.0	9.69	Mt^a,b^
GmGLYI-17	Glyma.12 g167400	Glyma.12 g167400.1	12	32196920–32200101	555	2	184	21.0	5.14	Cy^a,b^; Ec^b^
GmGLYI-18	Glyma.13 g106600	Glyma.13 g106600.1	13	22081599–22084230	630	5	209	23.4	6.49	Ch^a,c^; Ec^b^, Mt^b^
GmGLYI-19	Glyma.13 g168200	Glyma.13 g168200.1	13	28259866–28261852	504	3	167	19.0	5.46	Ec^a^; Cy^b^; Nu^b^
GmGLYI-20	Glyma.15 g009500	Glyma.15 g009500.1	15	737953–739806	522	3	173	19.4	5.58	Ch^a,b^; Cy^b^
GmGLYI-21	Glyma.15 g108400	Glyma.15 g108400.1	15	8534420–8539477	864	8	287	32.4	5.74	Cy^a,b^
GmGLYI-22	Glyma.16 g003500	Glyma.16 g003500.1	16	194687–196274	549	2	182	20.6	5.22	Cy^a,b^; Ch^b^
GmGLYI-23	Glyma.17 g052700	Glyma.17 g052700.1	17	4011185–4013445	621	5	206	22.9	7.08	Ch^a,c^; Mt^b^
GmGLYI-24	Glyma.17 g115900	Glyma.17 g115900.1	17	9163290–9164088	438	4	145	16.5	6.88	Ch^a^, Ec^a,b^; Nu^b^

Abbreviations: *CDS* coding DNA sequence, *Chro* chromosome, *PP* polypeptide length, *MW* molecular weight, *P* ^*I*^ isoelectric point, *bp* base pair, *aa* amino acid, *kDa* kilodalton, *Ch* chloroplast, *Cy* cytosol, *Ec* extracellular, *Mt* mitochondria, *Nu* nucleus, *Po* peroxisome

^a^Localization prediction by CELLO v.2.5 (http://cello.life.nctu.edu.tw/)

^b^Localization prediction by pSORT (http://www.genscript.com/wolf-psort.html)

^c^Chloroplast localization signal confirmed by ChloroP (http://www.cbs.dtu.dk/services/ChloroP/)

Similarly, soybean GLYII proteins have been primarily identified using a previously characterized *Brassica juncea* GLYII protein (GenBank: AAO26580.1) as query and secondarily by the newly identified members. A total of 26 unique protein sequences have been identified and checked for the presence of unique metallo-beta-lactamase domain (PF00753) using Pfam. Three of them didn’t have this unique domain and were discarded from the list. Thus, a total of 23 soybean GLYII proteins have been confirmed which is greater than the previously reported *Arabidopsis* (9) and rice (4) GLYII family members. These 23 GLYII proteins have been coded by 12 unique genes located on ten different chromosomes (Fig. 1). They were named as *GmGLYII*-1 to *GmGLYII*-12 like *GmGLYI* genes (Table 2). In both *GmGLYI* and *GmGLYII* families, the number of proteins was greater than the number of genes (Tables 1 and 2); indicating the existence of alternate splicing event in soybean glyoxalase genes. Most of the *GmGLYI* genes (17 out of 24) and *GmGLYII* genes (5 out of 12) showed only a single product. However, rest seven *GmGLYI* genes formed 24 alternative spliced products, whereas seven *GmGLYII* genes lead to the generation of 18 proteins (Tables 1 and 2).

**Table 2:** List of identified *GLYII* genes in Soybean (*Glycine max*) along with their detailed information and localization

Name	Gene	Protein	Chromosome no	CDS coordinate (5’ to 3’)	CDS (bp)	Exons	PP length (aa)	MW (kDa)	pI	Localization
GmGLYII-1	Glyma.02 g220100	Glyma.02 g220100.1	2	40797403–40800820	609	5	202	22.8	5.62	Cy^b^
GmGLYII-2	Glyma.04 g224100	Glyma.04 g224100.1	4	49456049–49460172	600	6	199	22.0	7.12	Cy^a,b^
		Glyma.04 g224100.2			546	6	181	20.0	6.54	Cy^a^; Ec^b^
		Glyma.04 g224100.3			486	5	161	17.9	6.49	Cy^a,b^
		Glyma.04 g224100.4			432	4	143	15.9	7.59	Cy^a^, Ec^b^
GmGLYII-3	Glyma.06 g140800	Glyma.06 g140800.1	6	11478165–11482810	777	7	258	28.7	6.86	Cy^b^
GmGLYII-4	Glyma.11 g126200	Glyma.11 g126200.1	11	9591802–9595913	948	7	315	34.7	6.06	Nu^a^; Mt^b^
		Glyma.11 g126200.2			861	7	286	31.5	5.92	Ch^a,b^; Mt^a,b^
GmGLYII-5	Glyma.12 g050800	Glyma.12 g050800.1	12	3651594–3655661	951	8	316	35.0	5.93	Ch^a^; Mt^b^
		Glyma.12 g050800.2			948	8	315	34.9	5.94	Nu^a^; Mt^b^
		Glyma.12 g050800.3			924	8	307	34.0	6.22	Nu^a^; Mt^b^
GmGLYII-6	Glyma.13 g261400	Glyma.13 g261400.1	13	36531853–36536326	1134	9	377	41.4	8.87	Ch^a^; PM^b^, Ec^b^
GmGLYII-7	Glyma.13 g345400	Glyma.13 g345400.1	13	43601121–43604841	990	7	329	36.3	8.82	Ch^a,c^; Mt^b^
		Glyma.13 g345400.2			876	8	291	32.0	7.71	Mt^a,b^
GmGLYII-8	Glyma.14 g187700	Glyma.14 g187700.1	14	45250598–45254278	777	7	258	28.7	5.65	Cy^a,b^
		Glyma.14 g187700.2			546	5	181	20.0	5.84	Cy^a,b^
GmGLYII-9	Glyma.15 g028900	Glyma.15 g028900.1	15	2325622–2329211	981	7	326	35.8	9.0	Ch^a,c^; Mt^b^
		Glyma.15 g028900.2			948	8	315	34.7	8.88	Ch^a,c^; Mt^b^
GmGLYII-10	Glyma.15 g245500	Glyma.15 g245500.1	15	46785385–46788016	570	5	189	20.8	9.03	Mt^b^; Ec^b^
GmGLYII-11	Glyma.18 g163500	Glyma.18 g163500.1	18	37294122–37294499	258	2	85	9.5	6.34	Cy^b^; Ec^b^
GmGLYII-12	Glyma.20 g118000	Glyma.20 g118000.1	20	36083279–36091841	1584	12	527	58.8	6.56	Ch^a^; Cy^b^
		Glyma.20 g118000.2			1467	12	488	54.5	5.92	Ch^a^; Cy^b^
		Glyma.20 g118000.3			1467	12	488	54.5	5.92	Ch^a^; Cy^b^

Abbreviations: *CDS* coding DNA sequence, *PP* polypeptide length, *MW* molecular weight, *P* ^*I*^ isoelectric point, *bp* base pair, *aa* amino acid, *kDa* kilodalton, *Ch* chloroplast, *Cy* cytosol, *Ec* extracellular, *Mt* mitochondria, *Nu* nucleus

^a^Localization prediction by CELLO v.2.5 (http://cello.life.nctu.edu.tw/)

^b^Localization prediction by pSORT (http://www.genscript.com/wolf-psort.html)

^c^Chloroplast localization signal confirmed by ChloroP (http://www.cbs.dtu.dk/services/ChloroP/)

### Detailed analysis of identified GmGLYI and GmGLYII members

All the newly identified GmGLYI and GmGLYII members were analyzed in detail. The coding DNA sequence (CDS) length of the *GmGLYI* members vary from 333 bp (*GmGLYI-12.1*) to 1101 bp (*GmGLYI-4.1*) with an average of 740 bp. Consequently, *GmGLYI-4.1* encodes for the largest protein of the family with a polypeptide length of 366 aa and molecular weight of 40.6 kDa; and the smallest protein (GmGLYI-12.1) is 110 aa in length with 12.8 kDa in weight (Table 1). Similar to the length and molecular weight variation, the proteins showed a wide range of deviation in their isoelectric point (pI) value from 4.86 (GmGLYI-9.1) to 9.69 (GmGLYI-16.3). Most of the GmGLYI members showed acidic pI value (less than or around 7), with only seven such as GmGLYI-1.3, GmGLYI-4.1, GmGLYI-10.5, GmGLYI-11.2, GmGLYI-15.1, GmGLYI-16.1, and GmGLYI-16.3 have showed basic pI value (Table 1). This ensures the presence of both positively and negatively charged GmGLYI proteins at a certain physiological condition. Sub-cellular localization of all these predicted GmGLYI proteins (41) were analyzed based on two different tools CELLO [23] and Wolf pSORT [24], and the chloroplast localization was further confirmed by ChloroP [25]. Different members were found to be localized at different sub-cellular compartments, such as chloroplast, cytosol, mitochondria, nucleus, extracellular, peroxisome. Most of the GmGLYI proteins are found to be localized in cytosol, followed by chloroplast, mitochondria and nucleus (Table 1).

Similarly, the CDS length of *GmGLYII* transcripts varies from 432 bp (*GmGLYII-2.4*) to 1584 bp (*GmGLYII-12.1*) with an average of 850 bp (Table 2). The largest GmGLYII-12.1 protein is 527 aa in length with a molecular weight of 58.8 kDa; and the smallest protein (GmGLYII-2.4) is 143 aa in length and 15.9 kDa in weight (Table 2). GmGLYII proteins also show variation in their pI values ranging from 5.62 (GmGLYII-1.1) to 9.03 (GmGLYII-10.1). Most of the GmGLYII members (15 out of 23) showed acidic pI value similar to GmGLYI proteins, while only eight GmGLYII members such as GmGLYII-2.1, GmGLYII-2.4, GmGLYII-6.1, GmGLYII-7.1, GmGLYII-7.2, GmGLYII-9.1, GmGLYII-9.2, and GmGLYII-10.1 have basic pI value (Table 2). Similar to GmGLYI, most of the GmGLYII proteins are found to be localized in cytosol, followed by chloroplast (4), nucleus (3), and mitochondria (2).

### Chromosomal distribution and gene duplication

To determine the exact position and distribution of the identified *GmGLYI* and *GmGLYII* genes on different chromosomes, a detailed chromosome map was constructed. Soybean glyoxalase genes are found to be unevenly distributed throughout the chromosomes. It has been found that 24 *GmGLYI* genes are located on 13 different chromosomes (Fig. 1a). The gene density per chromosome is highly uneven, where Chromosome 9 and 11 contain the maximum occurrence of *GLYI* genes (3 each). However, chromosomes 1, 7, 8, 12, 13, 15, 18 have two *GLYI* genes each, and only one *GLYI* gene each is present on chromosomes 4, 5, 6, and 16. No *GLYI* gene was found on chromosomes 2, 3, 10, 14, 18, 19 and 20; thereafter not shown in the Fig. 1a. Similarly, 12 *GmGLYII* genes were found to be located on ten different chromosomes (Fig. 1b) and the gene density per chromosome is highly uneven. Chromosomes 13 and 15 contain the maximum *GLYII* genes (2 each), whereas chromosomes 2, 4, 6, 11, 12, 14, 18, and 20 have only one *GLYII* gene each. No *GLYII* gene was found on the rest of the chromosomes and as such not shown in the Fig. 1b. All the *GmGLYI* and *GmGLYII* genes were found to be located towards the chromosome ends (Fig. 1), suggesting the possibility of inter-chromosomal genetic rearrangements between different soybean chromosomes during genome duplication.

Due to two duplication events, soybean genome resulted in many paralogs within a gene family [14]. Out of the 24 GmGLYI proteins (only the first member in case of different alternate splice form), 20 are clustered in pairs (10 pairs) and eight GmGLYII proteins are clustered in pairs (4 pairs) out of a total of 12 GmGLYII proteins in the phylogenetic tree (Additional file 1: Figure S1). The percentage of similarities between all these GmGLYI (Additional file 2: Table S1) and GmGLYII (Additional file 2: Table S2) proteins were combined separately. It was observed that all the paired members of both GLYI and GLYII family (GmGLYI-1/-11, GmGLYI-4/-8, GmGLYI-10/-21, GmGLYI-3/-5, GmGLYI-14/-15, GmGLYI-18/-23, GmGLYI-2/-13, GmGLYI-17/-22, GmGLYI-19/-24 and GmGLYI-6/-9; GmGLYII-4/-5, GmGLYII-9/-11, GmGLYII-2/-3, GmGLYII-6/-10) have very high level (more than 90 %) of sequence similarities. This high level of sequence similarities indicates the possibility of segmental duplication of the genes throughout evolution. Moreover, among the 24 *GmGLYI* genes one gene pair (*GmGLYI*-14 and *GmGLYI*-15) was present continuously (without any gene in between) within a distance of less than 5 kb (1200 bp exactly) on chromosome 11. This indicates that these two genes might be duplicated by tandem duplication (Fig. 1). To identify the time course of gene duplication, all the identified duplicated gene pairs were analyzed using plant genome duplication database (http://chibba.agtec.uga.edu/duplication/index/downloads) [26] (Table 3). According to the ratio of nonsynonymous to synonymous substitutions (Ka/Ks), the evolutionary history of selection acting on different genes could be measured [17, 27]. This ratio could be used to interpret the direction and magnitude of natural selection enforcing on the various protein coding genes. A pair of sequences having Ka/Ks < 1 implies purifying selection; Ka/Ks = 1 indicates both sequences are drifting neutrally; and lastly Ka/Ks > 1 implies positive or Darwinian selection [17, 28]. The Ka/Ks of 15 glyoxalase duplicated gene pairs (Table 3) was found to be less than 0.55; that indicates the influence of purifying selection in the evolution of these gene pairs. Considering the divergence rate of 6.161029 synonymous mutations per synonymous site per year for soybean [29], the duplication time for each gene pairs was calculated. It is observed that all the segmental duplicated pairs showed a time frame between 3.7 and 18.8 Mya, except the tandem duplicated pair that occurred 33.9 Mya ago (Table 3).

**Table 3:** Divergence time between glyoxalase gene pairs in Soybean

Sl. no	Locus 1	Locus 2	ka	ks	ka/ks	Duplication time (Mya)	Duplication type
1	GmGLYI-1	GmGLYI-11	0.0354	0.1246	0.2841	10.2131	Segmental
2	GmGLYI-2	GmGLYI-13	0.0066	0.0983	0.0671	8.0574	Segmental
3	GmGLYI-3	GmGLYI-5	0.0317	0.0823	0.3852	6.7459	Segmental
4	GmGLYI-4	GmGLYI-8	0.0451	0.1094	0.4122	8.9672	Segmental
5	GmGLYI-6	GmGLYI-9	0.0418	0.1581	0.2644	12.9590	Segmental
6	GmGLYI-10	GmGLYI-21	0.0076	0.0455	0.1670	3.7295	Segmental
7	GmGLYI-14	GmGLYI-15	.2260	0.4137	0.5463	33.9098	Tandem
8	GmGLYI-17	GmGLYI-22	0.0263	0.1434	0.1834	11.7541	Segmental
9	GmGLYI-18	GmGLYI-23	0.0839	0.1666	0.5036	13.6557	Segmental
10	GmGLYI-19	GmGLYI-24	0.0450	0.1442	0.3121	11.8197	Segmental
11	GmGLYII-1	GmGLYII-8	0.0804	0.1449	0.5549	11.8770	Segmental
12	GmGLYII-2	GmGLYII-3	0.1142	0.1502	0.7603	12.3115	Segmental
13	GmGLYII-4	GmGLYII-5	0.0278	0.1353	0.2055	11.0902	Segmental
14	GmGLYII-6	GmGLYII-10	0.0916	0.229	0.4000	18.7705	Segmental
15	GmGLYII-7	GmGLYII-9	0.0246	0.0767	0.3207	6.2869	Segmental

### Phylogenetic analysis of glyoxalase genes from various plant species

**Fig. 2:**
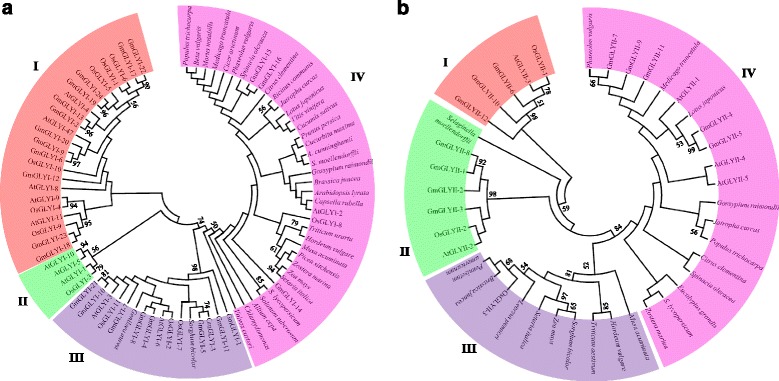
Phylogenetic analyses of GLYI (**a**) and GLYII (**b**) proteins from various plant species. Glyoxalase protein sequences from various plant species were downloaded from various databases and provided as Additional files 4 and 5. An unrooted tree was generated using Neighbor-Joining method with 1000 bootstrap by MEGA5.2 software using the full-length amino acid sequences of eighty-three GLYI (**a**) or forty-one GLYII (**b**) proteins (only the first splice variants were taken in case of multiple splice forms). The numbers next to the branch shows the result of 1000 bootstrap replicates expressed in percentage, and scores higher than 50 % are indicated on the nodes. Both trees were sub-divided into four classes (marked by I to IV) and indicated by different colours

In the present study, a phylogenetic tree of all the identified GmGLYI or GmGLYII proteins along with other reported GLYI or GLYII proteins from other plant species were constructed using Mega 5.2 tool (Fig. 2). A neighbour joining phylogenetic tree was generated using a total of 83 full-length GLYI protein sequences of soybean, rice and *Arabidopsis* GLYI family, and proteins from other plant species. The tree was sub-divided into four subfamilies (I to IV) as evident in Fig. 2a. All these subfamilies consist of representative member from both dicot *Arabidopsis* and monocot rice, indicating that the evolution of plant *GLYI* genes occurred before the split of dicot-monocot. Clade-IV has the largest GLYI members from different plant species, while clade-II has the lowest number of members only from *Arabidopsis* and rice genome (Fig. 2a). Clade-I comprises of GLYI members only from the complete genome database of three plants, *Arabidopsi*s, rice and soybean. Among them, OsGLYI-10 is functionally a diverge member of the rice GLYI family and might possess some other activities than GLYI (unpublished data). In clade-III, there are multiple members from *Arabidopsis*, rice and soybean; and one member each from *Genlisea aurea* and *Sorghum bicolor*. Among them, three rice members OsGLYI-2, OsGLYI-7 and OsGLYI-11; and two members of *Arabidopsis* AtGLYI-3 and AtGLYI-6 have been already predicted to be Ni^2+^-dependent GLYI enzyme [2]. Thus rest of the members of this clade would be expected to have Ni^2+^-dependent catalytic activity. Similarly, clade-IV has members from rice (OsGLYI-8) and *Arabidopsis* (AtGLYI-2) which are Zn^2+^-dependent GLYI enzymes [2]. Thus rest of the GLYI members from other species would require Zn^2+^ for their optimum GLYI activity. This indicates that Zn^2+^-dependent GLYI enzymes are more diverse as they are present in many plant species (Fig. 2a).

To clarify the phylogenetic relationship among GLYII proteins, we further constructed another tree for all full length sequences of GmGLYII, OsGLYII, AtGLYII family and GLYII sequences from other plant species (Fig. 2b). This tree was subdivided into four classes (I to IV) too like the previous one. Class-I consists of three proteins from soybean, and one each from rice (OsGLYII-1) and *Arabidopsis* (AtGLYII-3). Among them, OsGLYII-1 has been reported to have sulphur dioxygenase (SDO) activity rather than GLYII [11]. So this sub class of proteins would be functionally diverse from GLYII. Similarly, class-II contains one protein each from rice (OsGLYII-2), *Arabidopsis* (AtGLYII-2), and *Selaginella moellendorffii*, and four proteins from soybean. AtGLYII-2 has been reported to be the mitochondrial localized AtGLYII family member [30]. Division of class-III and –IV is more interesting and evolutionarily more significant. Class-III has GLYII proteins from all monocot plants (rice, *Zea mays*, *Pennisetum*, *Brassica*, *Triticum*, *Hordeum*); while class-IV has exclusively dicot members including *Arabidopsis*, soybean, *Medicago*, lotus etc. (Fig. 2b). Apart from GLYI, GLYII proteins were found to be diversified after the split of monocot and dicot.

### Gene structures of *GmGLYI* and *GmGLYII* genes

**Fig. 3:**
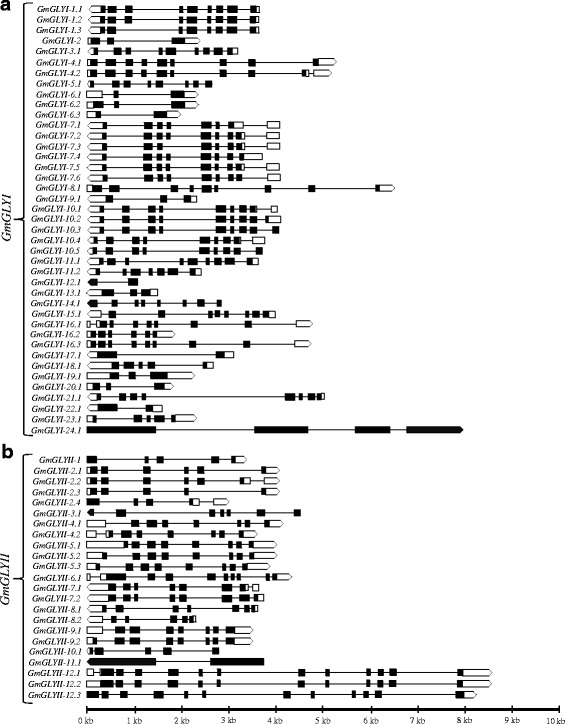
Gene structures of *GmGLYI* (**a**) and *GmGLYII* (**b**) family members including the alternative spliced forms. All the exons are shown in filled black boxes and the introns are indicated by black lines. The 5’-UTR regions are shown using empty boxes and the 3’-UTR regions are shown in empty arrows which also indicate the direction of the gene. Left to right direction of transcript indicates “+” strand, while the right to left one indicates “-” strand, relative to the annotation of the genome sequence. The size of the introns, exons, and UTRs could be estimated from the scale at the bottom

Detailed analysis of the exon-intron structure of *GmGLYI* (Fig. 3a) and *GmGLYII* (Fig. 3b) genes showed great variation among themselves. All *GmGLYI* and *GmGLYII* genes contained at least one intron in their open reading frame (ORF), which means there is no intron less glyoxalase gene in soybean. The number of introns varied from 1 to 9 in the ORFs of different *GmGLYI* genes (Fig. 3a and Additional file 3: Table S3). The *GmGLYI*-6.3, *GmGLYI*-12, *GmGLYI*-17, and *GmGLYI*-22 contained a single intron in their ORF while the largest numbers of introns (9) were found in the *GmGLYI*-4.2 transcript. In many cases, the borders of protein-coding sequence, 5′ and 3′ untranslated regions (UTR) also contain large numberof introns [13, 31]. Out of 41 *GmGLYI* transcripts, there was no intron in the 3′ UTR of any of these genes and only eight of them contained a single intron in their 5′ UTR region. Similarly, the number of introns varied from 1 to 12 in the ORFs of different *GmGLYII* genes (Fig. 3b and Additional file 3: Table S4). The maximum number of introns (12) was observed in *GmGLYII*-12.1, followed by 11 each in *GmGLYII*-12.2 and *GmGLYII*-12.3. *GmGLYII*-11.1 contained only a single intron in its ORF while the rest have varied number of introns. Similar to *GmGLYI* transcripts, there was no intron in the 3′ UTR of *GmGLYII* transcripts. Only six out of 23 transcripts (*GmGLYII*-2.2, *GmGLYII*-2.4, *GmGLYII*-4.2, *GmGLYII*-6.1, *GmGLYII*-7.1 and *GmGLYII*-12.1) have a single intron in their 5′UTR region.

Longer introns are selectively advantageous that could counterbalance the mutational bias and improve the recombination frequency [32]. A strong evidence for the presence of ancestral introns was reported by analyzing introns of animal, plant and fungus [33]. Moreover, the number of exons and introns were found to be similar in the paralogous genes (Fig. 3) that clustered together in the phylogenetic analysis (Additional file 1: Figure S1). Such as, *GmGLYI*-1/-11, *GmGLYI*-4/-8, *GmGLYI*-10/-21, and *GmGLYI*-6/-9 have the same number of introns and exons.

### Analysis of GmGLYI proteins for their domain architecture, catalytic conservance and metal ion dependency

**Fig. 4:**
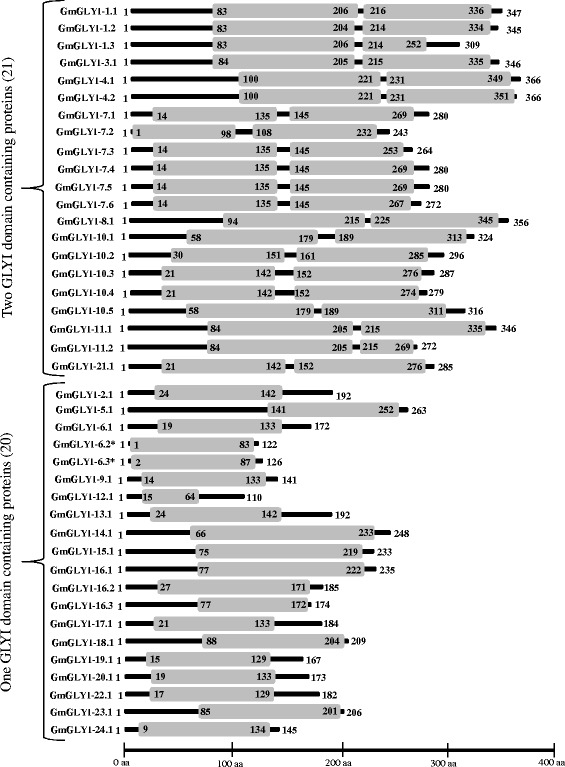
Domain architectures of GmGLYI proteins. All forty-one soybean GLYI proteins were analyzed for the presence of functional domain(s) using pfam (http://pfam.xfam.org/). All the GmGLYI proteins possess glyoxalase domain (PF00903) that is represented by boxes. The position of the domain(s) is indicated by the amino acid number inside the box. Among the 41 GmGLYI members, 20 of them have single glyoxalase domain, whereas rest 21 have two domains. The length of full proteins is indicated by exact amino acid numbers and relative position of the domains could be interpreted by the scale given below

All the predicted GmGLYI (41) proteins were analyzed using Pfam to reveal the presence of conserved glyoxalase domain (PF00903) among them. Analyses of GmGLYI proteins revealed that 21 out of forty-one contains two GLYI domains, while the rest 20 have only single GLYI domain (Fig. 4). Presence of two GLYI domain in a single protein have been previously reported from *Saccharomyces cerevisiae* [34], *Oryza sativa* [12] and *Plasmodium falciparum* [35]. Presence of two domain forms two putative active sites on a single monomeric protein. Both the active sites are found to be functional, but allosterically regulated in *Plasmodium falciparum* [35], whereas one of the active site is found to be a pseudo-active site in *Oryza sativa* [12]. However, GLYI proteins with single domain have also been reported from various species such as *E. coli* [36], *H. sapiens* [37] and function as homo-dimer.

**Fig. 5:**
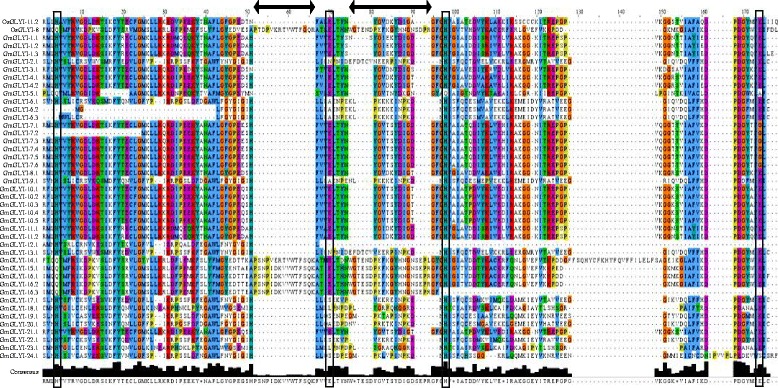
Multiple sequence alignment of GLYI domain of all GmGLYI proteins along with that of OsGLYI-11.2 and OsGLYI-8. GLYI domain (N-terminal one in case of two domain containing proteins) of all GmGLYI proteins were aligned with that of a Ni^2+^-dependent OsGLYI-11.2 and a Zn^2+^-dependent OsGLYI-8 using ClustalW program. The alignment file was viewed using Jalview multiple alignment editor program. All four conserved metal binding sites were represented as black boxes and the specific region for Zn^2+^-dependent GLYI was marked with black arrow

Activity of GLYI enzyme is highly dependent on divalent metal ions [2]. On the basis of metal ion specificity GLYI proteins could be divided into two classes; Zn^2+^-dependent or Zn^2+^-independent (mainly Ni^2+^/Co^2+^-dependent). GLYI from *Homo sapiens*, *Saccharomyces cerevisiae* and *Pseudomonas putida* have been reported as Zn^2+^-dependent [38–40], whereas GLYI from *E. coli* and one of the rice GLYI (OsGLYI-11.2) showed Ni^2+^-dependent activity [12, 36]. The metal dependency of the GLYI enzymes could be easily predicted from the length of GLYI domain, as Ni^2+^-dependent GLYI has a domain length of ~120 aa and Zn^2+^-dependent GLYIs are usually 142 aa in length [2]. Irrespective of the metal ion dependency, the active site of GLYI proteins has a conserved motif of H/QEH/QE. Among them, the glutamate residues act as a base by accepting protons from the substrate and any mutation of this conserved residue resulted in the complete loss of activity [12, 41]. Thus, to comment on the presence of enzymatic activity and metal ion dependency, GLYI domain (only N-terminal one in case of two domain containing members) of all the putative GmGLYI proteins were aligned (Fig. 5) along with known Ni^2+^-dependent OsGLYI-11.2 and Zn^2+^-dependent OsGLYI-8 [2] proteins. All the metal binding sites were presented inside black boxes and the regions specific for Zn^2+^-dependent GLYI were presented by black arrows (Fig. 5).

Based on the presence of all the four conserved metal binding site, the expected GLYI enzyme activity of the putative GmGLYI proteins was predicted (Table 4). Out of a total 41 putative GmGLYI proteins, 20 have all the four conserved residues and are expected to have functional GLYI enzyme activity (Fig. 5 and Table 4). Out of this 20 expected functional GLYI enzymes, 16 are predicted to be Ni^2+^-dependent as they have the domain length of around 120 aa and lack of the conserved regions specific for Zn^2+^-dependent members. The remaining four namely GmGLYI-14.1, GmGLYI-15.1, GmGLYI-16.1 and GmGLYI-16.2 are expected to be Zn^2+^-dependent as their domain length is more than 145 aa and possessed the conserved regions (Fig. 5 and Table 4).

**Table 4:** Analysis of all putative GmGLYI enzymes for their enzymatic activity and metal ion dependency

Sl. no	Proteins	Metal binding sites				Expected GLYI enzyme activity	Length of GLYI domain (aa)	Metal ion dependency
		H/Q	E	H/Q	E			
1	GmGLYI-1.1	√	√	√	√	Present	124	Ni
2	GmGLYI-1.2	√	√	√	√	Present	122	Ni
3	GmGLYI-1.3	√	√	√	√	Present	124	Ni
4	GmGLYI-2.1	√	√	√	√	Present	125	Ni
5	GmGLYI-3.1	√	√	√	√	Present	122	Ni
6	GmGLYI-4.1	√	√	√	√	Present	122	Ni
7	GmGLYI-4.2	√	√	√	√	Present	122	Ni
8	GmGLYI-5.1	√	√	√	—	Absent	115	-
9	GmGLYI-6.1	√	—	√	√	Absent	121	-
10	GmGLYI-6.2	—	—	√	√	Absent	83	-
11	GmGLYI-6.3	—	—	√	√	Absent	87	-
12	GmGLYI-7.1	√	√	√	—	Absent	122	-
13	GmGLYI-7.2	—	√	√	—	Absent	98	-
14	GmGLYI-7.3	√	√	√	—	Absent	122	-
15	GmGLYI-7.4	√	√	√	—	Absent	122	-
16	GmGLYI-7.5	√	√	√	—	Absent	122	-
17	GmGLYI-7.6	√	√	√	—	Absent	122	-
18	GmGLYI-8.1	√	√	√	√	Present	122	Ni
19	GmGLYI-9.1	√	—	√	√	Absent	120	-
20	GmGLYI-10.1	√	√	√	√	Present	122	Ni
21	GmGLYI-10.2	√	√	√	√	Present	122	Ni
22	GmGLYI-10.3	√	√	√	√	Present	122	Ni
23	GmGLYI-10.4	√	√	√	√	Present	122	Ni
24	GmGLYI-10.5	√	√	√	√	Present	122	Ni
25	GmGLYI-11.1	√	√	√	√	Present	122	Ni
26	GmGLYI-11.2	√	√	√	√	Present	122	Ni
27	GmGLYI-12.1	√	—	—	—	Absent	50	-
28	GmGLYI-13.1	√	—	√	√	Absent	125	-
29	GmGLYI-14.1	√	√	√	√	Present	169	Zn
30	GmGLYI-15.1	√	√	√	√	Present	145	Zn
31	GmGLYI-16.1	√	√	√	√	Present	146	Zn
32	GmGLYI-16.2	√	√	√	√	Present	145	Zn
33	GmGLYI-16.3	√	√	—	—	Absent	96	-
34	GmGLYI-17.1	√	—	√	√	Absent	119	-
35	GmGLYI-18.1	√	—	√	√	Absent	117	-
36	GmGLYI-19.1	√	—	√	√	Absent	121	-
37	GmGLYI-20.1	√	—	√	√	Absent	121	-
38	GmGLYI-21.1	√	√	√	√	Present	122	Ni
39	GmGLYI-22.1	√	—	√	√	Absent	119	-
40	GmGLYI-23.1	√	—	√	√	Absent	117	-
41	GmGLYI-24.1	√	—	√	—	Absent	126	-

### Analysis of GmGLYII proteins for their domain architecture and catalytic efficiency

**Fig. 6:**
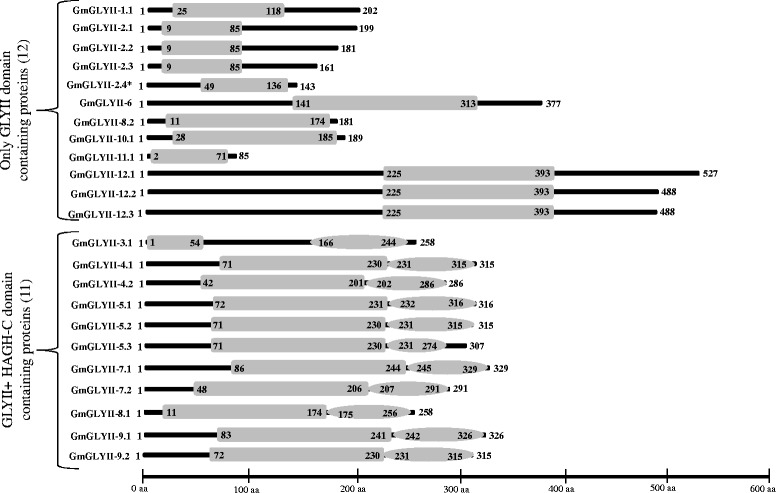
Domain architectures of GmGLYII proteins. All 23 soybean GLYII proteins were analyzed for the presence of functional domain(s) using pfam. There were two types of domains observed in GmGLYII proteins, such as β-lactamase domains (represented by box) and hydroxyacylglutathione hydrolase C-terminus (HAGH-C) domain (represented by circle). The length of full protein and domain(s) are indicated by exact amino acid numbers beside and inside of the shape, respectively. The relative size could be identified by using the scale below

**Fig. 7:**
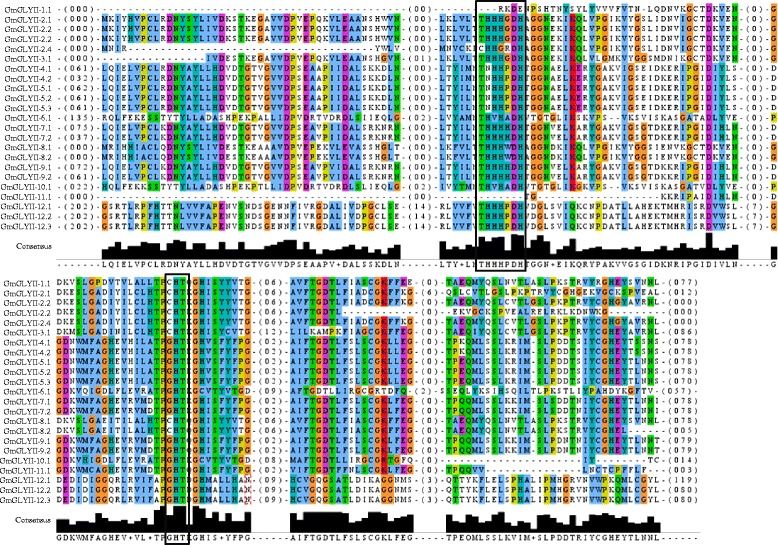
Multiple sequence alignment of GmGLYII proteins. All GmGLYII full length protein sequences were aligned using ClustalW program and viewed using Jalview multiple alignment editor program. The black boxes indicate the most conserved metal binding motif (THHHXDH) and active site motif (G/CHT)

Genome wide analysis of soybean revealed the presence of 23 GLYII proteins coded by 12 genes (Table 2). All these GmGLYII proteins were analyzed using Pfam to reveal the presence of conserved metallo-beta-lactamase domain (PF00753) among them. Analysis of all GmGLYII proteins revealed that 12 out of 23 have only metallo-beta-lactamase domain, while the rest eleven contain additional Hydroxyacylglutathione hydrolase C-terminus (HAGH-C) domain (PF16123) along with metallo-beta-lactamase domain (Fig. 6). HAGH-C domain is usually found to be present at the C-terminus of GLYII enzymes that forms the substrate binding site along with the catalytic domain (PF00753) [42]. However, GLYII from various species such as *E. coli, S. cerevisiae, S. typhimurium, L. infantum, A. thaliana, B. juncea, O. sativa* and *H. sapiens,* contained the well conserved metal binding motif (THXHXDH) and active site motif (C/GHT) [9]. Both these motifs play an important role in the GLYII enzyme activity of a protein. Therefore, to comment on the presence of enzymatic activity of the putative GmGLYII proteins, their protein sequences were aligned by multiple sequence alignment (Fig. 7). Both these motifs were indicated by black boxes (Fig. 7); their presence and absence were listed in Table 5. Out of 23 putative GmGLYII proteins only three of them do not possess the conserved metal binding residues, but all of them have the active site motif (Fig. 7 and Table 5). Thus, it could be expected that all the predicted GmGLYII proteins have the functional GLYII enzyme activity except GmGLYII-1.1, GmGLYII-2.4, and GmGLYII-11.1 (Table 5).

**Table 5:** Sequence analyses of all putative GmGLYII proteins for the presence of conserved motifs and enzyme activity

Sl. no	Proteins	Conserved metal binding motif (THHHXDH)	Active site motif (C/GHT)	Expected GLYII enzyme activity
1	GmGLYII-1.1	Absent	Present	No
2	GmGLYII-2.1	Present	Present	Yes
3	GmGLYII-2.2	Present	Present	Yes
4	GmGLYII-2.3	Present	Present	Yes
5	GmGLYII-2.4	Absent	Present	No
6	GmGLYII-3.1	Present	Present	Yes
7	GmGLYII-4.1	Present	Present	Yes
8	GmGLYII-4.2	Present	Present	Yes
9	GmGLYII-5.1	Present	Present	Yes
10	GmGLYII-5.2	Present	Present	Yes
11	GmGLYII-5.3	Present	Present	Yes
12	GmGLYII-6.1	Present	Present	Yes
13	GmGLYII-7.1	Present	Present	Yes
14	GmGLYII-7.2	Present	Present	Yes
15	GmGLYII-8.1	Present	Present	Yes
16	GmGLYII-8.2	Present	Present	Yes
17	GmGLYII-9.1	Present	Present	Yes
18	GmGLYII-9.2	Present	Present	Yes
19	GmGLYII-10.1	Present	Present	Yes
20	GmGLYII-11.1	Absent	Present	No
21	GmGLYII-12.1	Present	Present	Yes
22	GmGLYII-12.2	Present	Present	Yes
23	GmGLYII-12.3	Present	Present	Yes

### Homology modelling of representative GmGLYI and GmGLYII members

**Fig. 8:**
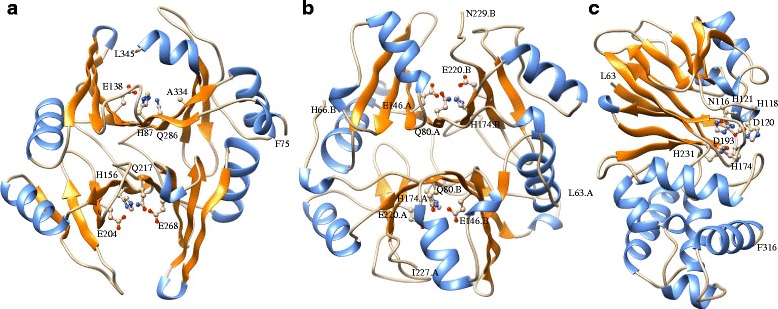
Three dimensional homology model structure of soybean glyoxalase proteins. Structures of GmGLYI-3 (**a**), GmGLYI-16 (**b**) and GmGLYII-5 (**c**) were built using Swiss-model server based on available close similar structure from Protein Data Bank (PDB) *Zea mays* GLYI (5D7Z), mouse GLYI (4OPN), and AtGLYII-2 (2Q42) proteins, respectively. All the α-helices were marked with orange colour, while β-sheets were marked with cornflower blue. The active sites residues were identified based on the alignment with template structure and shown by ball-stick model. The structure and active residues were visualized and generated using chimera program

To know the arrangement of active site residues and overall 3-D coordination, homology model of GmGLYI-3, GmGLYI-16 and GmGLYII-5 proteins was built (Fig. 8) based on the closely related template structure of *Zea mays* GLYI (PDB: 5D7Z) [43], mouse GLYI (PDB: 4OPN), and AtGLYII-2 (PDB: 2Q42) [30] proteins, respectively. GmGLYI-3 is a Ni^2+^-dependent monomeric GLYI enzyme (Fig. 8a), while GmGLYI-16 is a Zn^2+^-dependent homodimeric enzyme (Fig. 8b). GmGLYI-3 has two putative active sites; one consists of H-156, E-204, Q-217 and E-268, and the other one consists of H-87, E-138, Q-286 and A-334. The second putative active site has lack of a highly conserved Glu residues, thus might be inactive in nature like previously reported OsGLYI-11.2 [12]. The Zn^2+^-dependent GmGLYI-16 consists of single GLYI domain (Fig. 4) and thus forms homo-dimer to create two putative active sites (Fig. 8b). One putative active site has Q80 and E146 (from one chain, A) and H174 and E220 (from another chain, B); another one has opposite members from both chains. Here both the active sites have all four conserved residues and thus are predicted to be functionally active too. On the other hand, GmGLYII-5 is a monomeric protein consists of two structural orientations, an N-terminal domain (L63 to D193) with two βββαβ topology and a C-terminal domain (T194 to F316) with five α-helices (Fig. 8c). The metal binding and active site residues are Asn116, His118, Asp120, His121, His174, Asp193 and His231 (Fig. 8c) are found to be conserved as compared to template AtGLYII-2 protein.

### Expression analysis of *GmGLYI* and *GmGLYII* genes at different soybean tissues

**Fig. 9:**
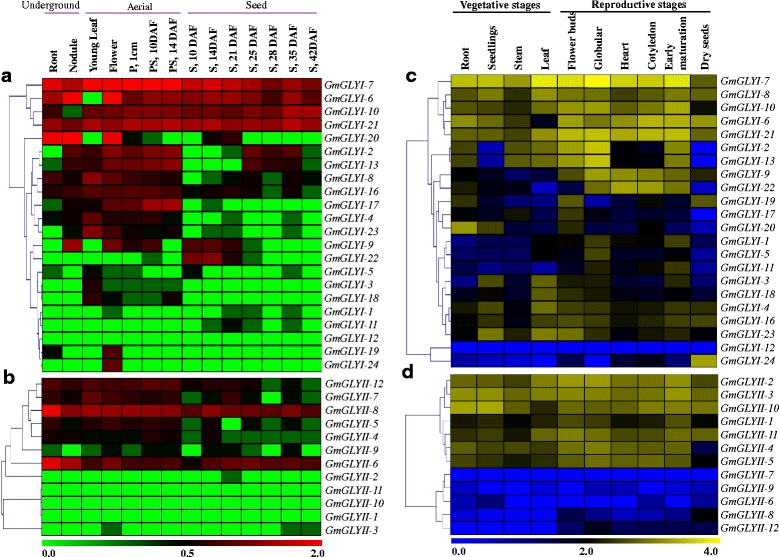
Expression profiling of Soybean glyoxalase genes with hierarchical clustering in different developmental tissues and stages. **a**, **b** RNA-seq expression data of 14 developmental tissues, such as R (root), N (nodule), YL (young leave), F (flower), P.1 cm (one cm pod), PS.10d (pod shell 10DAF), PS.14d (pod shell 14DAF), S.10d (seed 10DAF), S.14d (seed 14 DAF), S.21d (seed 21DAF), S.25d (seed 25DAF), S.28d (seed 28DAF), S.35d (seed 35DAF), S.42d (seed 42DAF) was used in the analysis. The normalized data was downloaded from soybase (http://soybase.org/soyseq/) and provided as Additional file 2: Table S5. Heatmap generation and hierarchical clustering was performed using MeV software package. The colour scale below the heat map indicates expression values; green indicates low transcript abundance while red indicates high level of transcript abundance. **c**, **d** Transcriptome data of all *GmGLYI* and *GmGLYII* genes at various developmental stages (indicated at the top of each lane) were obtained from the National Center for Biotechnology Information (http://www.ncbi.nlm.nih.gov/geo/query/acc.cgi?acc=GSE29163). Heatmaps generation and hierarchical clustering were performed using MeV software package. The colour scale given below the heat map indicates the expression values where blue indicates low transcript abundance and yellow indicates high transcript abundance

RNA-Seq Atlas of *Glycine max* provides high-resolution gene expression data in a diverse set of 14 soybean tissues such as young leaf, flower, one cm pod (7 days after flowering, DAF), pod-shell(10 DAF and 14 DAF), seed (10, 14, 21, 25, 28 and 35 DAF), root and nodule. All these tissues could be broadly divided into three classes; such as underground, aerial and seed. RNA-seq normalized expression data for all *GmGLYI* and *GmGLYII* genes were retrieved from soybase (http://www.soybase.org/soyseq/), except *GmGLYI-14* and *GmGLYI-15* due to lack of their appropriate probe (Additional file 2: Table S5). Data were analyzed and represented as heat maps generated using TIGR MeV software package (Fig. 9a and b). Expression analyses of all *GmGLYI* genes revealed that the different members have different tissue specific expression. Among all the 22 analyzed genes, *GmGLYI*-7 showed highest level of constitutive expression in all the tissues, followed by *GmGLYI*-21, *GmGLYI*-10 and *GmGLYI*-6. This high level of constitutive expression indicates their significant role at all these plant tissues (Fig. 9a). A cluster of genes showed medium to high level of expression in all the underground and aerial tissues only, followed by very low expression at the seed tissues. They are *GmGLYI*-2, *GmGLYI*-13, *GmGLYI*-17, *GmGLYI*-8, *GmGLYI*-4 and *GmGLYI*-16. Previous studies on rice and *Arabidopsis* showed the presence of highly seed specific *GLYI* genes such as *AtGLYI*-8, *OsGLYI*-3 and *OsGLYI*-10 [12]. Similarly, three of *GmGLYI* genes such as *GmGLYI*-1, *GmGLYI*-11 and *GmGLYI*-22 showed medium level of expression in different seed tissues only (Fig. 9a), indicating the evolutionary conservance for the presence of seed specific *GLYI* genes.

Expression analyses of *GmGLYII* genes indicate two clear clades (Fig. 9b). Out of 12 analyzed genes, five genes such as *GmGLYII*-1, *GmGLYII*-2, *GmGLYII*-3, *GmGLYII*-10 and *GmGLYII*-11 showed almost undetectable expression in all the tissues with few exceptions. Among others, *GmGLYII*-8 showed highest level of constitutive expression in all the tissues, followed by *GmGLYII*-6*.* These two genes might play a major role in all tissues. Similar to *GmGLYI*, a cluster of genes (*GmGLYII*-4, *GmGLYII*-5, *GmGLYII*-7, and *GmGLYII*-12) showed medium level of expression in the underground and aerial tissues, except the seed (Fig. 9b). No tissue specific expression pattern was observed in case of *GmGLYII* genes.

From the expression data analysis of the identified paralogous pairs of *GmGLYI* and *GmGLYII* genes in 14 soybean tissues revealed a high level of expression divergence. For example, *GmGLYI*-6 showed high level constitutive expression while its paralogous *GmGLYI*-9 showed detectable expression in a few tissues. However, some of the paralogous *GmGLYI* gene pairs namely *GmGLYI*-1/-11, *GmGLYI*-2/-13, *GmGLYI*-4/-8, *GmGLYI*-10/-21, and *GmGLYI*-19/-24 showed similar pattern of expression. The divergence is even more in case of *GmGLYII* gene pairs. For instance, *GmGLYII*-8 is highly expressed in all the analyzed tissues while its paralogous counterpart *GmGLYII*-1 remains mostly undetectable. Similar level of deviation was also observed in case of *GmGLYII*-6/-10and *GmGLYII*-7/-9 gene pairs.

### Expression analysis of *GmGLYI* and *GmGLYII* genes at different developmental stages

Expression of *GmGLYI* and *GmGLYII* genes at different developmental stages was analyzed using publicly-available genome-wide transcript profiling data of soybean (http://www.ncbi.nlm.nih.gov/geo/query/acc.cgi?acc=GSE29163). The dataset contains mainly two broad developmental sets, one at vegetative stages (roots, seedlings, stems, leaves) and the other at reproductive stage (floral buds, different stages of seed development- globular, heart, cotyledon, early-maturation, dry). As shown in Fig. 9c, most of the *GmGLYI* genes showed high level of expression, without any distinct pattern of expression. Out of the 24 *GmGLYI* genes, only *GmGLYI*-12 showed undetectable expression at all stages. Among others, *GmGLYI*-7 showed maximum constitutive expression in all the developmental stages followed by *GmGLYI*-21, *GmGLYI*-6, *GmGLYI*-10 and *GmGLYI*-8 (Fig. 9c). Two of *GmGLYI* members, *GmGLYI*-22 and *GmGLYI*-9 showed only reproductive stage specific expression. This indicates the development specific modulation of *GmGLYI* gene expression.

On the other hand, a distinct division is observed in the *GmGLYII* gene expression through the developmental stages (Fig. 9d). A cluster of genes such as *GmGLYII*-1, *GmGLYII*-2, *GmGLYII*-3, *GmGLYII*-6, *GmGLYII*-10, and *GmGLYII*-11 showed either undetectable or very low level of expression in both vegetative and reproductive phases. However, rest of the *GmGLYII* members showed medium to high level of expression in all the developmental stages constitutively (Fig. 9d). High level of expression of both *GmGLYI* and *GmGLYII* genes in all the developmental stages of soybean indicates the constitutive metabolic/cellular role of glyoxalase pathway throughout the life cycle of plant.

### Expression analysis of soybean glyoxalase genes under stress

**Fig. 10:**
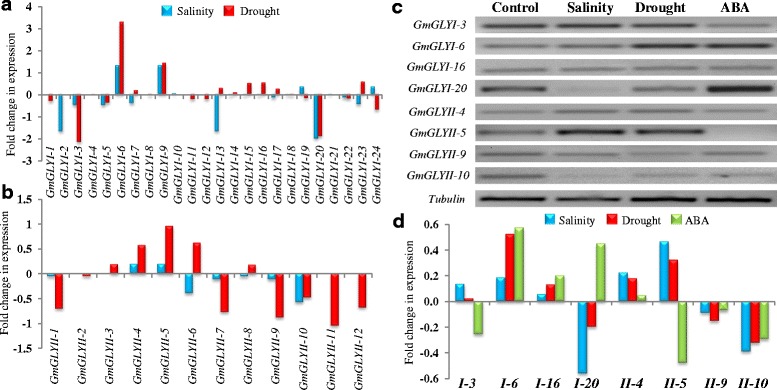
Expression analyses of soybean glyoxalase genes in response to salinity, drought and hormonal treatment. Relative expression data of all available *GmGLYI* (**a**) and *GmGLYII* (**b**) genes under salinity and drought stresses were obtained from the National Center for Biotechnology Information GEO database (http://www.ncbi.nlm.nih.gov/geo/). Expression data is presented as fold-change by comparing with the corresponding mock samples. Blue colour bar represents data for salinity stress, while red colour indicates drought stress. **c** Semiquantitative RT-PCR of four *GmGLYI* genes (*GmGLYI*-3, *GmGLYI*-6, *GmGLYI*-16 and *GmGLYI*-20), four *GmGLYII* genes (*GmGLYI*-4, *GmGLYI*-5, *GmGLYI-9* and *GmGLYI-10*) and one house-keeping control gene, *Tubulin* under different conditions such as control, salinity, drought and ABA treatment. **d** Relative expression analysis of the representative eight glyoxalase genes were analyzed by measuring the PCR band intensity using Image J software and represent as relative fold change in expression

To gain deep insights into the function of glyoxalase genes in the abiotic stress adaptation of soybean, the expression profiles of all *GmGLYI* and *GmGLYII* genes were analyzed in response to salinity and drought stresses using publicly available microarray data. Expression data sets were retrieved from gene expression omnibus database of GSE41125 and GSE40627 for salinity and drought stress, respectively. Data were not available for all these genes due to the limitation of respective probes. Among the total of 24 *GmGLYI* and 12 *GmGLYII* genes; data for 19 *GmGLYI* and eight *GmGLYII* genes were analyzed for salinity, while expression data of 21 *GmGLYI* and 12 *GmGLYII* genes were found for drought stress. Different glyoxalase members responded differentially in terms of their expression towards both these stresses (Fig. 10a and b). In response to salinity stress, four *GmGLYI* genes and two *GmGLYII* genes showed up-regulation, while seven *GmGLYI* genes and four *GmGLYII* genes showed down-regulation and rest of them remained unaltered (Fig. 10a and b). Similarly, drought stress causes upregulation of seven *GmGLYI* genes and five *GmGLYII* genes, and down-regulation of eight *GmGLYI* genes and six *GmGLYII* genes (Fig. 10a and b). Among 24 *GmGLYI* genes; *GmGLYI*-6 and *GmGLYI*-9 showed significant up-regulation, while other two of them (*GmGLYI*-3 and *GmGLYI*-10) showed down-regulation in both the stresses (Fig. 10a). In case of *GmGLYII*; *GmGLYII*-4 and *GmGLYII*-5 showed up-regulation, while *GmGLYII*-10 showed significant down-regulation in both salinity and drought stresses (Fig. 10b). Rest of the members of both *GmGLYI* and *GmGLYII* family showed variable expression pattern. This indicates diverse role of different glyoxalase members in the stress modulation pathways of soybean plant.

To screen the role of soybean glyoxalase genes in response to salinity, drought and hormonal treatment (Abscisic Acid, ABA), a semi quantitative RT-PCR was performed to validate four candidate *GmGLYI* genes (*GmGLYI-3*, −*6*,-*16* and −*20*) and four candidate *GmGLYII* genes (*GmGLYII-4*, −*5*,-*9* and −*10*) which were highly responsive in microarray data analysis (Fig. 10a and b). For this purpose, 15 days old soybean seedlings were subjected to normal water (as control), 200 mM NaCl (for salinity) or withdrawn of water (for drought) or 10 mM ABA (for hormonal treatment) for 8 h. Expression of all the candidate genes were compared with that of *Tubulin* (act as a house-keeping control gene) (Fig. 10c). Relative transcript abundance of all these transcripts was measured by scanning the gel image using Image J software relative fold change in expression was calculated considering *Tubulin* as internal control (Fig. 10d). It could be clearly inferred from Fig. 10d that *GmGLYI-*6, *GmGLYII-*4 and *GmGLYII-*5 showed strong up-regulation in response to both salinity and drought, while *GmGLYI-*20, *GmGLYII-*9 and *GmGLYII-*10 showed clear down-regulation (Fig. 10d). The remaining two members, *GmGLYI-*3 and *GmGLYI-*16 showed slight up/down regulation as compared to control sample. Overall, the pattern of expression of these eight candidate genes (Fig. 10c) was found to be almost similar to that of microarray data (Fig. 10a and b).

### Identification of cis-elements in the promoter region of soybean glyoxalase genes

**Fig. 11:**
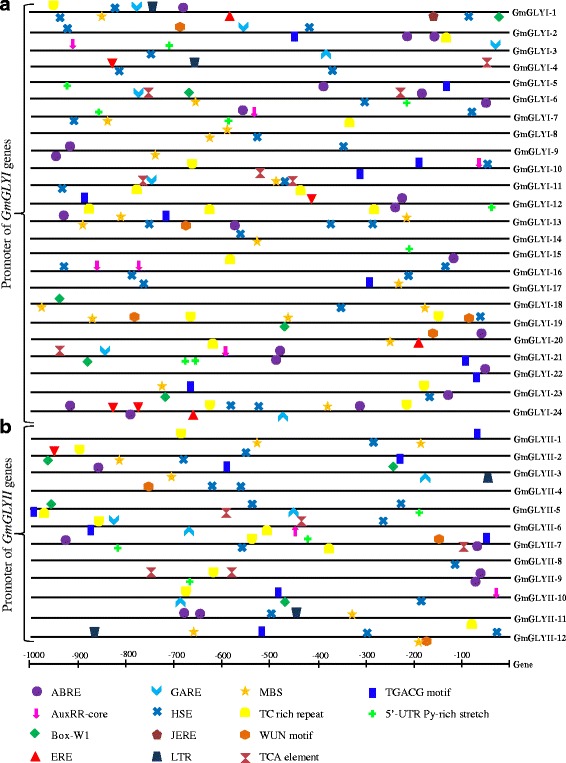
*In silico* promoter analysis of *GmGLYI* and *GmGLYII* genes. One kb 5’ upstream sequence of all *GmGLYI* and *GmGLYII* genes was downloaded from soybase database and scanned through PlantCARE for the identification of number and position of various cis-acting regulatory elements. Different regulatory elements were indicated by different colour symbols and placed in their relative position on the promoter. Symbols presented above the line indicate forward strand of DNA, while below one indicates the reverse strand

In order to comment on stress responsive expression of *GmGLY* genes in response to salinity, drought and ABA treatment, 1 kb upstream promoter region of each *GmGLYI* and *GmGLYII* genes were retrieved from soybase (http://www.soybase.org/dlpages/flank/index.php) and analyzed for the presence of cis-acting elements using PlantCARE [44]. This analysis leads to the identification of several stress-responsive cis elements such as abscisic acid responsive element (ABRE), auxin responsive element (AuxRR-core), fungal elicitor responsive element (BOX-W1), ethylene responsive element (ERE), gibberellin-responsive element (GARE), heat shock element (HSE), jasmonate and elicitor responsive element (JERE), low temperature responsive element (LTR), MYB-binding site (MBS), defence and stress responsive element (TC-rich), wounding and pathogen responsive elements (W-box and WUN-motif), salicylic acid responsive element (TCA), Methyl jasmonate-responsive element (CGTCA box and TGACG motif), element conferring high transcription level (5’ UTR Py-rich stretch). All these motifs are very crucial for plant stress modulation pathways and thus play important role to regulate the expression of various stress responsive genes [45, 46]. All these motifs were found to be distributed randomly in both the positive and negative strands of promoter sequences (Fig. 11). Among *GmGLYI* members, *GmGLYI-1* and *GmGLYI-24* have maximum cis-elements (12 elements), while *GmGLYI-14* promoter has minimum two elements on it. In case of *GmGLYII* members, *GmGLYII-7* has maximum ten elements, while *GmGLYII-8* has only one element. ABRE, HSE, and TGACG motif are found to be present in almost every promoter of *GmGLY* genes with few exceptions. Although correlation between the presence of cis-acting regulatory elements and the observed transcript abundance needs to be confirmed experimentally, these results indicated the stress-responsive nature of *GmGLY* genes.

## Discussion

Methylglyoxal (MG) is a metabolic by-product generated naturally in all living cells [7]. But the level of MG goes up in response to various abiotic stresses in plants [9]. It has been established in literature that glyoxalase pathway plays a vital role in the detoxification of MG as well as provides tolerance against multiple abiotic stresses [5, 7]. Genome wide analysis of glyoxalase pathway has been done preliminary in the monocot model plant rice and dicot model plant *Arabidopsis* [1]. However, this family has not been studied in any other species including legume. In the present study, we have performed a genome wide analysis of soybean to identify glyoxalase gene families, including their chromosomal location, gene and protein structure, conserved active site and catalytic site motifs and expression profiles. A total of 24 *GLYI* and 12 *GLYII* genes were identified in the soybean genome that codes for 41 GLYI and 24 GLYII proteins, respectively (Tables 1 and 2). The number of *GmGLYI* genes is 2.2 times more than that of *Arabidopsis* and rice (eleven genes each); and *GmGLYII* shows 2.4 times more abundance than that of *Arabidopsis* (five *AtGLYII* genes), and 4 times more abundant than rice (three *OsGLYII* genes). The possible reason behind this significant increase in gene number might be the two duplication events of soybean [14] that has occurred after the monocot/dicot split, or most of the soybean genes expanded in a species-specific manner [17].

To adopt with different adverse environmental conditions, plants tend to duplicate genes to generate novel members or increase number [17, 47]. There are three basic principal patterns of gene duplications, such as tandem duplication, segmental duplication and transposition. In the present analysis, a total of ten duplicated pairs were observed in *GmGLYI* family and five in *GmGLYII* family (Table 3). All of them showed segmental duplication except one, that is the major pattern of gene duplication in plant. The tandem duplication pair GmGLYI-14/-15 was formed 33.9 Mya ago; while the segmental duplications of *GmGLYI* genes have occurred between 3.7 and 13.6 Mya and that of *GmGLYII* occurred between 6.3 and18.8 Mya. This indicates that the tandem duplication event has occurred before the segmental duplication event. Similar pattern of duplication has been reported previously for HD-Zip genes of soybean [17].

Soybean, including other plants has been found to possess greater number of GLYI and GLYII genes and proteins as compared to their animal counterpart to date. One of the possible reasons behind this is gene duplication of plant during evolution that ultimately leads to functional divergence of genes [48]. Functional divergence might lead to either subfunctionalization or neofunctionalization, that in turn resulted in novel gene functions [48]. In the present study, out of 41 predicted GLYI proteins only twenty of them possess all four conserved metal binding sites and are expected to have functional GLYI enzyme activity (Fig. 5 and Table 4). The other proteins might be functionally diverged and possess some other activities similar to GLYI. Structurally GLYI is the member of vicinal oxygen chelate (VOC) superfamily that includes extradiol dioxygenases, GLYI and methylmalonyl‐CoA epimerase [49]. One of the earlier predicted GmGLYI (Accession no. X68819) was found to have Glutathione S-transferase activity, too [50]. Apart from that, it has been well-characterized in literature that there are two metal activation classes of GLYIs; Zn^2+^ and non-Zn^2+^ (mainly Ni^2+^/Co^2+^). Both these classes possess same four conserved metal binding residues and octahedral metal co-ordination; regardless of the metal activation class [51]. Three dimensional structure of one of the predicted Ni^2+^-dependent GLYI, GmGLYI-3 (Fig. 8a) and Zn^2+^-dependent GLYI, GmGLYI-16 (Fig. 8b) confirms the presence of same active site residues in both. However, Ni^2+^-dependent GmGLYI-3 found to be monomer consisting of two GLYI domains that fold to create two putative active sites (Fig. 8a). Whereas, single GLYI domain containing Zn^2+^-dependent GmGLYI-16 need to be homo- dimer to create two putative active sites (Fig. 8b). Interestingly, metal specificity of putative GLYIs could be easily predicted based on the protein’s amino acid length and sequence [2, 51]. Zn^2+^- activated GLYIs are relatively larger in amino acid length than Ni^2+^/Co^2+^-activated ones and have unique region in their sequence (Fig. 5). Based on these criteria, 16 out of 20 predicted functional GmGLYI enzymes are expected be Ni^2+^/Co^2+^-activated. Same pattern of dominance by Ni^2+^/Co^2+^-activated forms was observed in case of rice GLYIs (3 out of 4 expected active OsGLYIs) and *Arabidopsis* GLYIs (two out of three expected functional AtGLYIs) [2].

On contrary, GLYII enzymes contain the β-lactamase fold structure that includes lactonase, rubredoxin:oxygen oxidoreductase, GLYII, arylsulfatase, phosphodiesterase, carboxylesterase and tRNA maturase [52]. Previously, GLYII family members from *Arabidopsis* (AtGlx2-5) and rice (OsGLYII-1) have been reported to lack of GLYII activity, instead they possess sulphur dioxygenase like ethylmalonic encephalopathy protein 1 (ETHE1) activity [11]. Similar to GLYI, three out of 23 predicted GmGLYII proteins did not possess the conserved metal binding motif that might have resulted in the absence of GLYII activity and leads to the functional divergence.

Expression of glyoxalase genes has been found to be highly specific towards certain tissue or developmental stages in *Arabidopsis* and rice [1]. Thus, the expression pattern of *GmGLYI* and *GmGLYI* genes was analyzed at different developmental stages and tissues (Fig. 9). These data revealed the tissue specific expression pattern of glyoxalase genes in soybean too. Out of all, *GmGLYI*-7, *GmGLYI*-21 and *GmGLYII*-8 are found to be the constitutively expressed members of soybean glyoxalase system. A cluster of *GmGLYI* and *GmGLYII* genes maintained high level of expression in all the underground and aerial tissues, followed by low level of expression in the different stages of seed development (Fig. 9). This indicates the presence of functional distribution among the multiple members in different tissue/developmental stimuli. On contrary, some of the *GmGLYI* genes such as *GmGLYI*-1, *GmGLYI*-11, and *GmGLYI*-22 showed medium level of expression in the seed tissues only, with low or no expression in other parts (Fig. 9). This indicates development specific transition/regulation of *GmGLYI* genes. In case of *GmGLYII* genes, two distinguishable clades were observed in their expression where one set showed high level expression in all the tissues and another have no expression at all. These low expressive genes might have other cellular/metabolic regulation expect developmental/tissue regulation. Another interesting expression pattern was observed for soybean glyoxalase genes under abiotic stresses (Fig. 10). Different members of *GmGLYI* and *GmGLYII* families responded differentially against salinity, drought and hormone (ABA) treatment (Fig. 10c). *GmGLYII*-9 and *GmGLYII*-10 showed strong down-regulation in response to all three conditions, while *GmGLYII*-16 showed sharp up-regulation (Fig. 10d). Presence of various cis-acting regulatory elements on the putative promoter sequence of *GmGLYI* and *GmGLYII* genes might be a probable reason behind this altered expression (Fig. 11). Similar pattern of expression was observed previously in rice and *Arabidopsis* glyoxalase genes [1], where each member shows specific pattern of expression towards the particular type of stress treatment. Overall, the observed information in the present study will facilitate to find out the appropriate candidate gene(s) for further functional characterization and raising stress-tolerant transgenic crop plants.

## Conclusions

Taken together, we have performed a comprehensive *in silico* analysis of soybean glyoxalase gene families (*GmGLYI* and *GmGLYII*), and provided detailed information about them. Specifically, our results show that soybean genome contains 24 *GmGLYI* and 12 *GmGLYII* genes that code for 41 GmGLYI and twenty-three GmGLYII proteins, respectively; the largest identified glyoxalase gene family to date in any species/organism. Present study indicates genome-wide duplication (both segmental and tandem) of glyoxalase genes that lead to the expansion of family. Based on the presence of conserved motifs and sequence homology, we have provided insight into their putative function and metal dependency. Finally, expression data confirms the development, tissue and stress specific response of each and every gene in spite of having large multi-member family.

## Methods

### Identification of *GmGLYI* and *GmGLYII* genes in soybean

The putative GLYI and GLYII proteins in soybean genome were identified by BLASTP search against the new soybean genome database (Wm82.a2.v1) (http://www.soybase.org/) [53] with an e-value of 1 using previously reported soybean GLYI protein sequence (GenBank: NM_001249223.1) and *Brassica juncea* GLYII protein sequence (GenBank: AAO26580.1) as query, respectively. Subsequently, each of the identified sequences was used as secondary queries to find other new members. All the protein sequences were checked individually using Pfam (http://pfam.xfam.org/) with default parameters and e-value of 1, for the presence of glyoxalase domain (PF00903) in GLYI proteins and metallo-beta-lactamase domain (PF00753) in the GLYII proteins. All the identified putative glyoxalase proteins were nomenclature as prefix “Gm” for *Glycine max*, followed by GLYI or GLYII and Arabic numbers serially starting from 1 depending on their chromosomal position. Alternate splice forms were represented by adding arabic numbers after “.” sign sequentially. The chromosomal locations for all the putative *GmGLYI* and *GmGLYII* genes were identified from soybase (http://soybase.org/gb2/gbrowse/gmax1.01/) [53] database to draw the chromosomal map. Various physio-chemical properties of all the identified GmGLYI and GmGLYII proteins were calculated using Prot-Param software (http://web.expasy.org/protparam/). Localization of proteins were predicted using CELLO v.2.5: sub-cellular localization predictor (http://cello.life.nctu.edu.tw/) [23] and pSORT prediction software (http://www.genscript.com/wolf-psort.html) [24]. Chloroplast localization was further confirmed by ChloroP (http://www.cbs.dtu.dk/services/ChloroP/) [25].

### Multiple sequence alignment and phylogenetic analysis

To investigate the phylogenetic relationship and conserved motifs/metal binding sites among GLYI and GLYII proteins from various plant species, sequences were downloaded from NCBI (http://www.ncbi.nlm.nih.gov/), PDB (http://www.rcsb.org/pdb/home/home.do), rice genome database (http://rice.plantbiology.msu.edu/), *Arabidopsis* genome database (https://www.arabidopsis.org/) and soybean database (http://www.soybase.org/). Protein sequences used in the study of phylogenetic analysis were available in Additional files 4 and 5. Multiple sequence alignment was performed using ClustalW [54] and phylogenetic tree was constructed using MEGA 5.2 [55] with Neighbour-Joining method and 1000 bootstrap replicates.

### Gene duplication and Ka/Ks calculation

Gene duplication was analyzed using plant genome duplication database (http://chibba.agtec.uga.edu/duplication/index/downloads) [26] for soybean. Genes having more than 90 % sequence similarities were considered as segmental duplication, while tandem duplicated genes were separated by five or fewer genes in a 100-kb region. Synonymous (Ks) and nonsynonymous substitution (Ka) rates were retrieved from plant genome duplication database or calculated from PAL2NAL program (http://www.bork.embl.de/pal2nal/) [56]. Divergence time (in millions of years) was calculated for each gene pair considering a rate of 6.1X10^−9^ substitutions per site per year [17]. Thus, divergence time (T) = Ks/(2X6.1X10^−9^)X10^−6^ Mya.

### Assessment of domain architecture, catalytic conservance and metal ion specificity of GLYI and GLYII proteins

All the predicted GmGLYI (41) and GmGLYII (24) proteins were analyzed using Pfam to reveal the presence of conserved glyoxalase domain (PF00903) and metallo-beta-lactamase domain (PF00753), respectively. Glyoxalase domain (PF00903) of GmGLYI and metallo-beta-lactamase domain (PF00753) of GmGLYII proteins were aligned separately with previously characterized members using ClustalW and analyzed for the presence of conserved motifs. GLYI has a conserved H/QEH/QE motif for metal binding and catalysis, whereas GLYII has two separate metal binding motif (THXHXDH) and active site motif (C/GHT). The metal ion specificity of GmGLYI proteins was predicted based on the previous studies [2, 51].

### Homology based structural modelling of various soybean glyoxalase proteins

Homology based model of GmGLYI-3, GmGLYI-16 and GmGLYII-5 was built using SWISSMODEL program (http://swissmodel.expasy.org/) [57]. Respective protein sequences were first analyzed by template search, followed by model building using best template structure with highest similarities. Structures of GmGLYI-3, GmGLYI-16 and GmGLYII-5 were built using most similar structure available from Protein Data Bank (PDB) i.e. *Zea mays* GLYI (5D7Z), mouse GLYI (4OPN), and AtGLYII-2 (2Q42) proteins, respectively. Resulting structures were visualized using UCSF Chimera (http://www.cgl.ucsf.edu/chimera) [58]. Active site residues were identified and marked based on previous template structure analysis.

### Expression analysis using RNA-Seq Atlas of *Glycine max*

To analyze the tissue-specific expression data of 24 *GmGLYI* and 12 *GmGLYII* genes, their corresponding probe sets were indentified using soybase tool (http://www.soybase.org/correspondence/index.php). Normalized transcript data was downloaded from soybase (http://soybase.org/soyseq/) for 14 different tissues, including root, nodule (underground tissues); leaf, flower, pod-shell 10-day after flowering (DAF), pod-shell 14-DAF, one-cm pod (aerial tissues); and different stages of seed development (seed of 10-DAF, 14-DAF, 21-DAF, 25-DAF, 28-DAF, 35-DAF and 42-DAF). This normalized expression was used to generate heatmap and hierarchical clustering using the Institute for Genomic Research MeV software package [59].

### Expression analysis of *GmGLYI* and *GmGLYII* genes at different developmental stages

Expression patterns of *GmGLYI* and *GmGLYII* genes at different developmental stages were determined using the publically available transcriptomes data (http://www.ncbi.nlm.nih.gov/geo/query/acc.cgi?acc=GSE29163). Transcript data of ten different soybean stages (root, seedlings, stem, leaf, flower buds, and different stages of seed development- globular, heart, cotyledon, early maturation, and dry seeds) were downloaded from the NCBI database (http://www.ncbi.nlm.nih.gov/) with accession numbers of SRX062325 to SRX062334. After normalization, the values were used for heatmap generation using the Institute for Genomic Research MeV software package [59].

### Expression analysis of *GmGLYI* and *GmGLYII* genes in response to salinity and drought stresses

Expression data of glyoxalase genes in response to salinity and drought stresses were retrieved from the National Center for Biotechnology Information Gene Expression Omnibus (GEO) database [60] with accession numbers GSE41125 and GSE40627, respectively. Corresponding probe sets for *GmGLYI* and *GmGLYII* genes were identified using NetAffx Analysis Center (http://www.affymetrix.com/analysis/index.affx?navMode=cat530006&aId=netaffxNav) online Probe Match tool. More than one gene with the same probe set, were considered as same transcriptional profile, while in case of gene having more than one probe set, the highest value was considered. Expression data were normalized using that of mock, and represented as bar diagram.

### Plant material, stress treatments and semiquantitative RT-PCR

Soybean (*Glycine max L.* variety Sohag) seedlings were grown in a condition with continuous 30 °C temperature and 12 h/12 h of photoperiod [17]. Fifteen days old seedlings were irrigated with normal water as experimental control or 200 mM NaCl solution for salinity stress or 10 mM ABA solution for hormonal treatment for 8 h. Seedlings were placed onto filter paper and exposed to the air to mimic drought stress. Leaves were collected from all these seedlings after 8 h (with triplicates) and total RNA was extracted using TRIzol® Reagent (Thermo Fisher Scientific, USA). First-strand cDNA was synthesized using RevertAid First Strand cDNA Synthesis Kit (Thermo Fisher Scientific, USA). Gene-specific primer for eight candidates genes, listed in Additional file 3: Table S6, were designed using Primer-Blast (http://www.ncbi.nlm.nih.gov/tools/primer-blast/), and soybean *Tubulin* gene was used as an internal control [22].

### Promoter sequence analysis for putative cis-regulatory elements

To identify various cis-acting regulatory elements in the promoter sequences of *GmGLYI* and *GmGLYII* genes, 1 kb 5′ upstream region sequences were retrieved from soybean genome database (http://www.soybase.org/dlpages/flank/index.php). Promoter sequences were analyzed using PlantCARE databases [44] to find out the presence of cis-acting regulatory elements.

## Availability of data and materials

All sequence information regarding soybean is available at a public database, Soybase (http://soybase.org/). Apart from that, most datasets supporting the conclusions of this article are included as additional files. All protein sequences used in the phylogenetic analysis had been already deposited in Uniprot (http://www.uniprot.org/) and provided as additional data too. The seeds of Soybean (*Glycine max L.* variety Sohag) are available from Bangladesh Agriculture Research Institute, Gazipur, Bangladesh.

## Additional files

Additional file 1: Figure S1. Phylogenetic relationship of GmGLYI (A) and GmGLYII (B) proteins. An unrooted tree was generated using Neighbor-Joining method with 1000 bootstrap by MEGA5.2 software using the full-length amino acid sequences of the twenty-four GmGLYI and twelve GmGLYII proteins (only the first splice variants were taken in case of multiple members). The numbers next to the branch shows the result of 1000 bootstrap replicates expressed in percentage, and scores higher than 50 % are indicated on the nodes. (PDF 17 kb) Additional file 2: Table S1. Pairwise identities between paralogous pairs of GLYI proteins from Soybean. **Table S2.** Percentage of identities between all GLYII proteins from Soybean. **Table S5.** Expression analysis of soybean *GLYI* and *GLYII* genes through RNA-seq data (XLS 40 kb) Additional file 3: Table S3. Number of exons and introns in all the splice variants of *GmGLYI* genes. **Table S4.** Number of exons and introns in all the splice variants of *GmGLYII* genes. **Table S6.** Primers used in the semi-quantitative RT-PCR. (DOCX 26 kb) Additional file 4: Protein sequences used for phylogenetic analysis of GLYI. (DOCX 21 kb) Additional file 5: Protein sequences used for phylogenetic analysis of GLYII. (DOCX 17 kb)

## Abbreviations

aa: amino acid
ABA: abscisic acid
bp: base pair
DAF: day after flowering
GLYI: Glyoxalase I
GLYII: Glyoxalase II
GSH: reduced glutathione
h: hour
MG: methylglyoxal
Mya: million years
