# Supplementary material for: Genome-wide analysis and expression profiling of glyoxalase gene families in soybean (Glycine max) indicate their development and abiotic stress specific response
Source: BMC Plant Biol. 2016 Apr 16;16:87. doi: 10.1186/s12870-016-0773-9 (PMC4833937; doi:10.1186/s12870-016-0773-9)
Supplement: Additional file 1: Figure S1. — Phylogenetic relationship of GmGLYI (A) and GmGLYII (B) proteins. An unrooted tree was generated using Neighbor-Joining method with 1000 bootstrap by MEGA5.2 software using the full-length amino acid sequences of the twenty-four GmGLYI and twelve GmGLYII proteins (only the first splice variants were taken in case of multiple members). The numbers next to the branch shows the result of 1000 bootstrap replicates expressed in percentage, and scores higher than 50 % are indicated on the nodes. (PDF 17 kb) [file 12870_2016_773_MOESM1_ESM.pdf]

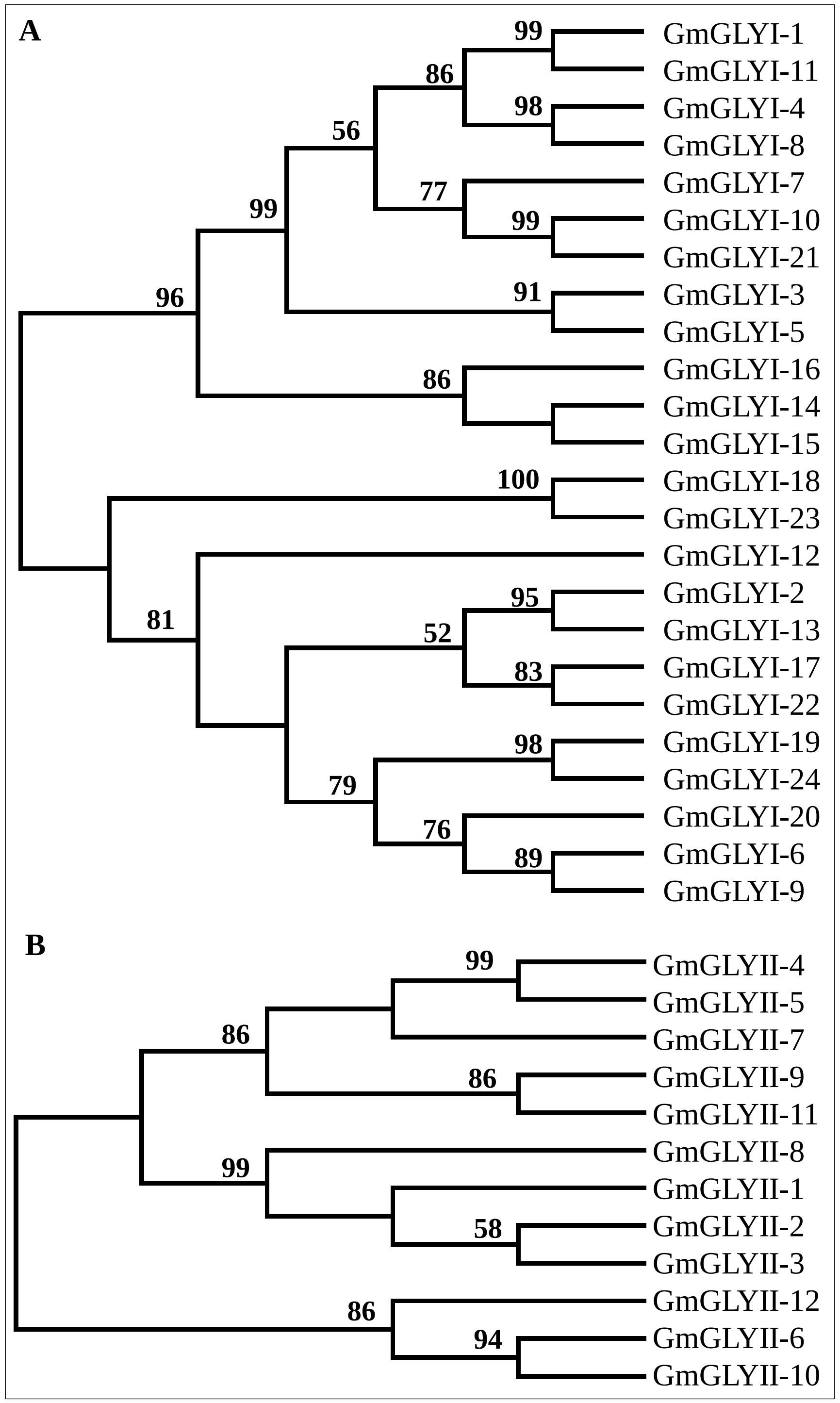

Figure S1 Phylogenetic relationship of GmGLYI (A) and GmGLYII (B) proteins. An unrooted tree was generated using Neighbor-Joining method with 1000 bootstrap by MEGA5.2 software using the full-length amino acid sequences of the twenty-four GmGLYI and twelve GmGLYII proteins (only the first splice variants were taken in case of multiple members). The numbers next to the branch shows the result of 1000 bootstrap replicates expressed in percentage, and scores higher than 50% are indicated on the nodes.
