# Supplementary material for: Genome-wide analysis and expression profiling of glyoxalase gene families in soybean (Glycine max) indicate their development and abiotic stress specific response
Source: BMC Plant Biol. 2016 Apr 16;16:87. doi: 10.1186/s12870-016-0773-9 (PMC4833937; doi:10.1186/s12870-016-0773-9)
Supplement: Additional file 3: Table S3. — Number of exons and introns in all the splice variants of GmGLYI genes. Table S4. Number of exons and introns in all the splice variants of GmGLYII genes. Table S6. Primers used in the semi-quantitative RT-PCR. (DOCX 26 kb) [file 12870_2016_773_MOESM3_ESM.docx]

| **Table S3 Number of exons and introns in all the splice variants of *GmGLYI* genes** | | | | | |
| --- | --- | --- | --- | --- | --- |
| Gene | No. of exons | No. of introns | No. of introns | | |
|  |  |  | CDS | 5'UTR | 3'UTR |
| *GmGLY1-1.1* | 9 | 8 | 8 | 0 | 0 |
| *GmGLY1-1.2* | 9 | 8 | 8 | 0 | 0 |
| *GmGLY1-1.3* | 8 | 7 | 7 | 0 | 0 |
| *GmGLY1-2* | 3 | 2 | 2 | 0 | 0 |
| *GmGLY1-3* | 9 | 8 | 8 | 0 | 0 |
| *GmGLY1-4.1* | 9 | 8 | 8 | 0 | 0 |
| *GmGLY1-4.2* | 9 | 9 | 8 | 1 | 0 |
| *GmGLY1-5* | 8 | 7 | 7 | 0 | 0 |
| *GmGLY1-6.1* | 2 | 2 | 2 | 0 | 0 |
| *GmGLY1-6.2* | 3 | 2 | 2 | 0 | 0 |
| *GmGLY1-6.3* | 2 | 1 | 1 | 0 | 0 |
| *GmGLY1-7.1* | 7 | 7 | 6 | 1 | 0 |
| *GmGLYI-7.2* | 8 | 8 | 7 | 1 | 0 |
| *GmGLYI-7.3* | 7 | 7 | 6 | 1 | 0 |
| *GmGLYI-7.4* | 8 | 7 | 7 | 0 | 0 |
| *GmGLY1-7.5* | 8 | 8 | 7 | 1 | 0 |
| *GmGLYI-7.6* | 8 | 8 | 8 | 0 | 0 |
| *GmGLYI-8* | 9 | 8 | 8 | 0 | 0 |
| *GmGLYI-9* | 3 | 2 | 2 | 0 | 0 |
| *GmGLYI-10.1* | 8 | 8 | 7 | 1 | 0 |
| *GmGLYI-10.2* | 9 | 8 | 8 | 0 | 0 |
| *GmGLYI-10.3* | 9 | 8 | 8 | 0 | 0 |
| *GmGLYI-10.4* | 8 | 8 | 7 | 1 | 0 |
| *GmGLYI-10.5* | 9 | 8 | 8 | 0 | 0 |
| *GmGLYI-11.1* | 9 | 8 | 8 | 0 | 0 |
| *GmGLYI-11.2* | 7 | 6 | 6 | 0 | 0 |
| *GmGLYI-12* | 2 | 1 | 1 | 0 | 0 |
| *GmGLYI-13* | 3 | 2 | 2 | 0 | 0 |
| *GmGLYI-14* | 8 | 7 | 7 | 0 | 0 |
| *GmGLYI-15* | 8 | 8 | 8 | 0 | 0 |
| *GmGLYI-16.1* | 7 | 8 | 7 | 1 | 0 |
| *GmGLYI-16.2* | 6 | 5 | 5 | 0 | 0 |
| *GmGLYI-16.3* | 8 | 8 | 8 | 0 | 0 |
| *GmGLYI-17* | 2 | 1 | 1 | 0 | 0 |
| *GmGLYI-18* | 5 | 4 | 4 | 0 | 0 |
| *GmGLYI-19* | 3 | 2 | 2 | 0 | 0 |
| *GmGLYI-20* | 3 | 2 | 2 | 0 | 0 |
| *GmGLYI-21* | 8 | 7 | 7 | 0 | 0 |
| *GmGLYI-22* | 2 | 1 | 1 | 0 | 0 |
| *GmGLYI-23* | 5 | 4 | 4 | 0 | 0 |
| *GmGLYI-24* | 4 | 3 | 3 | 0 | 0 |

CDS, protein-coding sequence; UTR, untranslated region

| **Table S4 Number of exons and introns in all the splice variants of *GmGLYII* genes** | | | | | |
| --- | --- | --- | --- | --- | --- |
| Gene | No. of exons | No. of introns | No. of introns | | |
|  |  |  | CDS | 5'UTR | 3'UTR |
| *GmGLY1I-1.1* | 5 | 4 | 4 | 0 | 0 |
| *GmGLYI1-2.1* | 6 | 5 | 5 | 0 | 0 |
| *GmGLYI1-2.2* | 6 | 6 | 5 | 1 | 0 |
| *GmGLYI1-2.3* | 5 | 4 | 4 | 0 | 0 |
| *GmGLY1I-2.4* | 4 | 4 | 3 | 1 | 0 |
| *GmGLYI1-3* | 7 | 6 | 6 | 0 | 0 |
| *GmGLY1I-4.1* | 7 | 7 | 7 | 0 | 0 |
| *GmGLYI1-4.2* | 7 | 7 | 6 | 1 | 0 |
| *GmGLYI1-5.1* | 8 | 7 | 7 | 0 | 0 |
| *GmGLYI1-5.2* | 8 | 7 | 7 | 0 | 0 |
| *GmGLYI1-5.3* | 8 | 7 | 7 | 0 | 0 |
| *GmGLYI1-6.1* | 9 | 9 | 8 | 1 | 0 |
| *GmGLYII-7.1* | 7 | 7 | 6 | 1 | 0 |
| *GmGLYII-7.2* | 8 | 7 | 7 | 0 | 0 |
| *GmGLYII-8.1* | 7 | 6 | 6 | 0 | 0 |
| *GmGLY1I-8.2* | 5 | 5 | 5 | 0 | 0 |
| *GmGLYII-9.1* | 7 | 7 | 7 | 0 | 0 |
| *GmGLYII-9.2* | 8 | 7 | 7 | 0 | 0 |
| *GmGLYII-10.1* | 5 | 4 | 4 | 0 | 0 |
| *GmGLYII-11.1* | 2 | 1 | 1 | 0 | 0 |
| *GmGLYII-12.1* | 12 | 12 | 11 | 1 | 0 |
| *GmGLYII-12.2* | 12 | 11 | 11 | 0 | 0 |
| *GmGLYII-12.3* | 12 | 11 | 11 | 0 | 0 |

CDS, protein-coding sequence; UTR, untranslated region

| **Table S6 Primers used in the semi-quantitative RT-PCR** | | | |
| --- | --- | --- | --- |
| **Sl. No** | **Primer name** | **Sequence (5’-3’)** | **Product size** |
| 1 | GmGLYI-3_FOR | GGAACCCGGGCCTGTTAAA | 212 bp |
|  | GmGLYI-3_REV | ACTTCTGCTCGGGGTTGTC |  |
| 2 | GmGLYI-6_FOR | GACTATGTGCGAGCAACGG | 141 bp |
|  | GmGLYI-6_REV | TGATCTTGCCACCTCACCA |  |
| 3 | GmGLYI-16_FOR | TCCGATTCCGATTCCGATCA | 143 bp |
|  | GmGLYI-16_REV | GCTCACTTTGGGGTCCTTGA |  |
| 4 | GmGLYI-20_FOR | ACGTGCATGCAACGGTAGA | 124 bp |
|  | GmGLYI-20_REV | CTCGCTGCTAAGGGGATCA |  |
| 5 | GmGLYII-4_FOR | AAGGGCTTGCCCACGATT | 131 bp |
|  | GmGLYII-4_REV | CCCAGGGCTTCTGCATCAT |  |
| 6 | GmGLYII-5_FOR | TTCCGAAGCTGCGCCTAT | 217 bp |
|  | GmGLYII-5_REV | ATGGCCTGCGAACATCCA |  |
| 7 | GmGLYII-9_FOR | ACACTCCTGGTCACACCCA | 127 bp |
|  | GmGLYII-9_REV | AGCATCTGCTGTGGGGTTC |  |
| 8 | GmGLYII-10_FOR | TGCCTCGCACCCAGAAAA | 210 bp |
|  | GmGLYII-10_REV | ACCGTTGCACCACTTGCT |  |
| 9 | GmTUBULIN_FOR | AACCTCCTCCTCATCGTACT | ~400 bp |
|  | GmTUBULIN_REV | GACAGCATCAGCATGTTCA |  |
