# Supplementary material for: Genome-wide analysis and expression profiling of glyoxalase gene families in soybean (Glycine max) indicate their development and abiotic stress specific response
Source: BMC Plant Biol. 2016 Apr 16;16:87. doi: 10.1186/s12870-016-0773-9 (PMC4833937; doi:10.1186/s12870-016-0773-9)
Supplement: Additional file 4: — Protein sequences used for phylogenetic analysis of GLYI. (DOCX 21 kb) [file 12870_2016_773_MOESM4_ESM.docx]

>Phaseolus vulgaris

MATSASIHRLSRLRFIANSQPLLSHFSIPSYFSLTPKTKKPNRLRFFSMAAEPKESPANNPGLHATPDEATKGYIMQQTMFRIKDPKVSLDFYSRVLGMSLLKRLDFPEMKFSLYFLGYEDTSKAPSNPVDKTVWTFSQKATIELTHNWGTESDPEFKGYHNGNSEPRGFGHIGITVDDTVKACERFQNLGVEFVKKPDDGKMKGIAFIKDPDGYWIEIFDQKTIGSVTQTAS

>Lotus japonicus

MAAEPKESPANNPGLHETPDEATKGYFMQQTMYRIKDPKVSLDFYSRILGMSLLKRLDFPEMKFSLYFMGYEDTTAAPSNSIDRTVWTFSQKATIELTHNWGTESDPEFKGYHNGNSEPRGFGHIGITVNDTYKACERFQNLGVEFVKKPDDGKMKGIAFIKDPDGYWIELFDRKSIGAVTQTA

>Cicer arietinum

MAASESKESPANNPGLHTTIDEATKGYFMQQTMFRIKDPKVSLDFYSRVLGMSLLKRLDFPEMKFSLYFMGYEDTTEAPSNPVDRTVWTFAQKATIELTHNWGTESDPEFKGYHNGNSDPRGFGHIGITVDDTYKACERFQNLGVEFVKKPDDGKMKGIAFIKDPDGYWIELFDRKTIGNVTEGNA

>Medicago truncatula

MAASESKESPANNPGLHATVDEATKGYFMQQTMFRIKDPKVSLDFYSRVLGMSLLKRLDFPEMKFSLYFMGYEDTSEAPSNSVDRTVWTFAQKATIELTHNWGTESDPEFKGYHNGNSDPRGFGHIGITVDDTYKACERFQNLGVEFVKKPEDGKMKGIAFIKDPDGYWIEIFDRKTIGNVTGSAA

>Vitis vinifera

MASCSIATSLSRLSLLRLIPKPSSSYSSSIPLFPTTTRKDPSRFRLFSASMASEPKESPSNNPGLHSSPDEATKGYFMQQTMYRIKDPKVSLDFYSRVLGMSLLKRLDFPEMKFSLYFMGYEDTASAPSNETERIVWTFSQKATIELTHNWGTESDPDFKGYHNGNSEPRGFGHIGITVDDTYKACERFERLGVEFVKKPDDGKMKGLAFIKDPDGYWIEIFDLRRIGTVSTTAA

>Morus notabilis

MAYSSITTTFSRLSLLRFASKSSSFSSSFPSLPFLDANSSPKVLNRFRVFSMASEPKELPANNPGLHTTPDEATKGYFMQQTMFRIKDPKVSLDFYSRVLGMSLLKRLDFPEMKFSLYFLGYEDPASAPANAVDRTVWTFGQKATIELTHNWGTESDPEFKGYHNGNSDPRGFGHIGITVDDTYKACERFERLGVEFVKKPDDGKLKGIAFIKDPDGYWIEIFDLKTIGQITASAS

>Prunus persica

MAIGRGGGDERTDRLCEQTAPALLCFHFSHKAPKYRFRLFSMASEPKESPANNPGLHATPDEATKSYFMQQTMFRIKDPKASLDFYSRVLGMSLLKRLDFPEMKFSLYFLGYEDPASAPTNPADRTVWTFGQKATIELTHNWGTESDPEFKGYHNGNSEPRGFGHIGVTVDDTYKACERFEHLGVEFVKKPDDGKMKGIAFIKDPDGYWVEIFDLKTIGSITGGAS

>Cucurbita maxima

MASAPKESPANNPGLHATPDDATKGYMMQQTMFRIKDPKASLDFYSRVLGMSLLKRLDFPDMKFSLYFLGYEDVASAPDNAVDRTVWTFGRKATIELTHNWGTESDPEFKGYHNGNSDPRGFGHIGITVDDTYKACERFERLGVEFVKKPDDGKMKGIAFIKDPDGYWIEIFDLKLIGNVTTNAA

>Ricinus communis

MATAATLSSLLSRFTLIKPPIISQSSRRIQSILRYSKLKNPNRLRLFSMASSEPKESPANNPGLYTTPDDATKGYIMQQTMYRIKDPKQSLDFYSHVLGMSLLKRLDFPEMKFSLYFMGYENTASAPTDPVERTVWTFGQKATIELTHNWGTESDPDFKGYHNGNSEPRGFGHIGITVDDVYKACERFKSLGVEFVKKPEDGKMKGIAFIKDPDGYWIEIFDLKTIGKTTGSAA

>Cucumis sativus

MASSPKESPANNPGLQATPDDATKGYIMQQTMYRIKDPKVSLDFYSRVLGMSLLKRLDFPEMKFSLYFMGYEDTASAPESSVDRTVWTFGRKATIELTHNWGTESDPEFKGYHNGNSDPRGFGHIGITVDDTIKACERFERLGVEFVKKPDDGKMKGIAFIKDPDGYWIEIFDLKTVGKVTSEAA

>Populus trichocarpa

MASEAKESASNNPGLHTTPDEATKGYIMQQTMFRIKDPKVSLDFYSRVLGMSLLKRLDFPEMKFSLYFLGYEDHASAPGDSVERTVWTFGRKATIELTHNWGTESDPEFKYHNGNSEPRGFGHIGVTVDDTYKACERFERLGVEFVKKPEDGKMKGIAFIKDPDGYWIEIFDLKTIGKITESAA

>Musa acuminata

MAAASLISHRLSSSFLRFSWRSASIPNSFAAPTQKLDRFRPFCSSMAASSSEPKEAPSNNPGLHTQLDDATKGYFLQQTMFRVKDPKVSLDFYSRVLGMSLLKRLDFPEMKFSLYFLGYENTSLAPSNPTERTVWTFGQKATIELTHNWGTENDPEFKGYHNGNSEPRGFGHIGITVDDTYKACERFERLGVEFVKKPDDGKMKGIAFIKDPDGYWIEIFDLNRIGNVTADAA

>Jatropha curcas

MASEPKESPANNPGLHTTIDEATKGYIMQQTMYRIKDPKISLDFYSRVLGMSLLKRLDFPDMKFSLYFLGYEDTTSAPNDPVERTVWTFGRKATIELTHNWGTESDPDFKGYHNGNSDPRGFGHIGITVDDVYKACERFEHLGVEFAKKPDDGKMKGIAFIKDPDGYWIEIFDLKTIGKTVSSAA

>Beta vulgaris

MASSVPKESSSNNPGLQATPDDATKGYIMQQTMFRIKDPKVSLDFYSRVLGMSLLKRLDFPEMKFSLYFMGYEDPASVPSDPVDRTVWTFSKKATIELTHNWGSESDPEFKGYHNGNTEPRGFGHIGITVDDTYKACERFEHLGVEFVKKPADGKMKGIAFIKDPDGYWIEIFDLKTIGNVAADAS

>Spinacia oleracea

MAASSEHKESPSNNPGLHSTPDDATKGYIMQQTMFRIKDPKVSLDFYSRVLGMSLLKRLDFPEMKFSLYFLGYEDPASAPTEPADRTVWTFSKKATIELTHNWGTESDPEFKGYHNGNSEPRGFGHIGVTVDDTFKACERFEQLGVEFVKKPDDGKMKGIAFIKDPDGYWIEIFDLKTIKSVAAGAS

>Allium cepa

MAASSQPKESPANNPGLQSEPDEATKGYFFQQTMFRIKDPKASLDFYSRVLGMSLLKRLDFPEMKFSLYFMGYENPQTAPADPTERTVWTFGQKATLELTHNWGTESDPEFKGYHNGNTDPRGFGHIGVTVDDAYKACERFESLGVEFVKKPDDGKMKGIAFIKDPDGYWIEIFDLKRIGNVTTAAA

>Solanum lycopersicum

MASESKDSPSNNPGLHATPDEATKGYFLQQTMFRIKDPKVSLEFYSKVLGMSLLKRLDFPEMKFSLYFMGYEDTASAPSDPVERTAWTFSQKSTLELTHNWGTESDPNFTGYHNGNSEPRGFGHIGVTVDDVYKACERFESLGVEFVKKPLDGKMKGIAFIKDPDGYWIEIFDTKIIKDAAGSAS

>Citrus clementina

MASSEPKESPANNPGLHTARDEATNGYFMQQTMFRIKDPKVSLDFYSRVLGMSLLKRLDFPEMKFSLYFLGYEDTASAPADPVDRTVWTFGKPATIELTHNWGTESDPDFKGYHNGNSEPRGFGHIGITVDDVYKACERFERLGVEFAKKPDGGKLKGVAFIKDPDDYWIEIFDLKTIGKIGGST

>Solanum tuberosum

MAASLPRAAFSLLSLLNSTINKPSLSPFSSPTPSFFTIKPKVTNFSRFISSSMASDSKDSPSNNPGLHATPDEATKGYFLQQTMFRIKDPKVSLEFYSKVLGMSLLKRFDFPEMKFSLYFMGYEDTTSAPSDPVERTAWTFSQKATLELTHNWGTESDPNFTGYHNGNSEPRGFGHIGVTVDDVYKACERFESLGVEFVKKPLDGKMKGIAFIKDPDGYWIEIFDTKLIKDTAGSAS

>Araucaria cunninghamii

MARARAMAMSLLSSAYPARLSRCPGGFSAANFGHSFHHPKSIKKQSLTTFATMATTVKESPANNPGLQDKPDEATKGYFMQQTMFRIKDPKASLDFYSRVLGMKLLKRLDFPDMKFSLYFLGYEDSDAAPSDPVERTVWTFGQKATIELTHNWGTESDPEFKGYHNGNSEPRGFGHIGITVDDTYKACERFEKLGVEFQKRPDDGKMKGIAFIKDPDGYWIEIFDLKRIGQVASHAS

>Brassica juncea

MASEAKESPANNPGLSTVRDEATKGYIMQQTMFRVKDPKASLDFYSRVLGMSLLKRLDFSEMKFSLYFLGYEDTSTAPTDPTERTVWTFGRPATIELTHNWGTESDPEFKGYHNGNSEPRGFGHIGVTVDDVHKACERFEQLGVEFVKKPHDGKMKNIAFIKDPDGYWIEIFDLKTIGTTAGNAA

>Triticum urartu

MSTSSGAKEAPANNPGLQAEADPATKGYIMQQTMFRVKDPKVSLDFYSRVMGMSLLKRLDFPEMKFSLYFLGYEDLSAAPADPVQRTGWTFGQKATIELTHNWGTESDPEFKGYHNGNSDPRGFGHIGVTVDDVYKACERFESLGVEFVKKPDDGKMKGIAFIKDPDGYWIEIFDLKRIGEVTAAAS

>Arabidopsis lyrata

MGSYSIAIAISRISPLIRFVKPYSTGFSFITCPCNSTRRPKRFDQLRVFSMASEARESPANNPGLSTVRDEATKGYIMQQTMFRIKDPKASLDFYSHVLGMSLLKRLDFSEMKFSLYFLGYEDTTTAPADPTERTVWTFGRPATIELTHNWGTESDPEFKGYHNGNSEPRGFGHIGVTVDDVHKACERFEELGVEFVKKPNDGKMKNIAFIKDPDGYWIEIFDLKTIGTTTVNAA

>Hordeum vulgare

MPAASLLRSSLLLSSHALRRLSSASASAPRGLRLAQPKGFGRSYPPALAAAAMSTSSGAKEAPANNPGLQAEVDPATKGYFMQQTMFRVKDPKVSLDFYSRVMGMSLLKRLDFPEMKFSLYFLGYEDLSAAPVDPVQRTGWTFGQKATIELTHNWGTENDPEFKGYHNGNSDPRGFGHIGVTVDDVYKACERFERLGVEFVKKPDDGKMKGIAFIKDPDGYWIEIFDLKRIGEVTGTAS

>Gossypium raimondii

MASSDLKESAANNPGLHTTPDEATKDYIMQQTMFRIKDPKASLDFYSRVLGMSLLKRVDVPELKFTLYFMGYEDVSKAPSDPYGRTVWTFGRAATIELTHNWGTESDPEFKGYHTGNSEPRGFGHIGITVDDVNKACERFERLGVEFVKKLDAGKMKGIAFIKDPDGYWIEIFDLKTIGDVISRCS

>Picea sitchensis

MAVVISAARLLLPIRHSRLLLSTCPPAPFAVSAANTSSESGHAFLAPKFITSFDQKQHHRLRAFATMASQAKEIPANNPGLNDKPDEATKGYFLQQTMLRVKDPKISLDFYSRVLGMKLLKRLDFPDMKFSLYFMGYEDTDAAPADSAERTVWTFQKVVIELTHNWGTESDPDFKGYHNGNSEPRGFGHFGITVDDTYKACERFAKLGVEFVKKPDDGKMKGLAFIKDPDGYWIEIFDLKRIGQITSSEA

>Zostera marina

MVAVAATFSFLTSPLLIKPRISSTFTATVLRSGKLRRFGRSRSFCCFPMASLKSKELPENNPGLCTQSDDATSNYFLQQTMLRVKDPKVSLDFYSRVLGMKLLKRLDFSEMKFSLYFLGYEDLALAPADPVERTIWTFSKKSTIELTHNWGTESDPEFKGHHNGNSEPRGFGHIGITVDDVYKACERFENLGVEFVKKPDAGKMKGIAFIKDPDGYWIEIFDLNLVGEVTRTAS

>Selaginella moellendorffii

MATTASKESAENNPGLSESPDEATKGYIVQQTMYRIKDPKASLDFYSRVLGMTLLKRLDFPDSKFSLYFVGYEDSAEAPKDPIERVRWTFRKKATIELTHNWGTETDPDFKGYHNGNADPRGYGHIGISVDDTYRACERFEKLGVEFVKKPDDGSMKGLAFIKDPDGYWIEIFDAGRIGGIVAGSA

>Zea mays

MATSSGPKEAPANNPGLQTEVDPATKGYFLQQTMLRVKDPKVSLDFYSRVMGMSLLKRLDFEEMKFSLYFLGYEDVTLAPDDHIKRTEWTFRQKATLELTHNWGTENDPEFKGYHNGNSDPRGFGHIGVTVDDVHKACERFERLGVEFVKKPDDGKIKGIAFIKDPDGYWIEIFDQTIGTVTSSAS

>Setaria italica

MAAASLLFPACALLRRLPAAPHLSRSARIKGFDRVRRFSPAAMSTSSGPKEAPGNNPGLHTEIDPATKGYFLQQTMLRVKDPKVSLDFYSRVMGMSLLKRLDFEELKFSLYFLGYEDVTSAPADHIKRTEWTFRQKATLELTHNWGTENDPEFKCYHNGNSDPRGFGHIGVTVDDVYKACERFERLGVDFVKKPDDGKIKGIAFIKDPDGYWIEIFDHTIGTVTASAS

>Chlamydomonas reinhardtii

MSGSLSELHAIPGVCVNPDPSTQNFVFQQTMFRIRDPAKSLDFYTRVLGMRLLAKLDFPDMKFSLYFLGYEDVKDIPEDPADRTVWMFRRKACLELTHNWGTESDPNFAGYHSGNSDPRGFGHIGFSVPDVYAACKRFEELGVEFQKKPDDGKMKGIAFIKDPDGYWIEILNADNARQFVGWGK

>Volvox carteri

MASLAELQSTPGVCANPDPATQGFVFQQTMYRIRDPVKSLDFYTRVLGMRLLSKLDFSDMKFSLYFLGYEDLKDIPEDAGDRTVWMFRRKACLELTHNWGTESDPAFAGYHNGNSDPRGYGHIGISVPDVYAACKRFEELGVEFQKRPDDGKMKGLAFIRDPDGYWIEVLNADNSRQFATWGK

>Capsella rubella

MSTHSLASAITRISPLIRFVKPYSTDFSFINCACNSASRRKRFDQLRVFSMASEARESSANNPGLSAIRDEATKGYIMQQTMFRIKDPKASLDFYSRVLGMSLLKRLDFSEMKFSLYFLGYEDTTTAPTDPTERTVWTFGRPATIELTHNWGTESDPEFTGYHNGNSEPRGFGYVLGEL

>Genlisea aurea

IIPMGSSIKPSLTSLAFTDPSCRRFNPLHVANATKKLQNSGLQTCKLFRGTIGSISASSTGGAAQASTATTQENFVDWVKKDNRRMLHVVYRVGDLDRTIKFYTECLGMKLLRKRDIPEERYTNAFLGYGSEDSHFVIELTYNYGVDKYDIGSGFGHFGIAVDDVAKTVDLIKAKGGNVIREPGPVKGGNTVIAFIEDPDGYKFELLERSPTPEPLCQVMLRVGDLDRSIEFYEKAYGMELLRKRDNPEYKYTIAMMGYGPEDKNAVVELTYNYGVTRYDRGNAYAQIAIGTDDVYRTAEGIRVYGGTITREPGPLPGINTKITACLDPDGWKTVFVDNVDFTKELE

>Sorghum bicolor

MARLLLPLPFAAAAAASASSLHLAASRLRLPAVSVARRECLYGGRVVGGVVRAPARLGKRGLCAGAEAGGSAGTVVGQEEALEWVKKDRRRLLHVVYRVGDLDKTIKFYTECLGMKLLRKRDIPEERYTNAFLGYGPEDSHFVVELTYNYGVESYDIGTAFGHFGIAVDDVAKTVELIKAKGGTVTREPGPVKGGKSVIAFIEDPDGYKFELIERGPTPEPLCQVMLRVGDLDRAINFYEKAFGMELLRKRDNPEYKYTIAMMGYGPEDKNAVLELTYNYGVKEYDKGNAYAQIAISTDDVYKTAEAIRVNGGRITREPGPLPGINTKITACTDPDGWKTVFVDNIDFLKELEE

>OsGLYI-1

MVNTTAGVKCGGGGAALPLSTLNHVSLVCRSLSTSLTFYRDFLGFVSVRRPGSFDFDGAWLFNYGIGIHLLQAEDPESMPPNKEINPKDNHISFTCESMEAVQRRLKEMGVRYVQRRVEEGGVYVDQIFFHDPDGFMIEICTCDKLPVVPLDAAAAHSIFAGRSPPPPVACKIRPVKQPSATKLGSVAAGGCVGEVIVVDAINGAAAAGGGGAMS

>OsglyI2

MRALPMAAGRAAAVAACASPAVPRRSLLLSTAAAGEPPCRPPADSSSPSKFSRFDRSAVRLLGWTAALQPEPVRLTRGASAAPKLRASPPDAAQAAAAFGSKEEAFAWAKSDNRRLLHVVYRVGDIDRTIKFYTECLGMKLLRKRDIPEEKYTNAFLGYGAEDNHFVVELTYNYGVDKYDIGAGFGHFGIAVDDVAKTVELIRAKGGKVTREPGPVKGGKTVIAFVEDPDGYKFEILERPGTPEPLCQVMLRVGNLDRAISFYEKACGMELLRKRDNPEYKYTVAMMGYGPEDKNAVLELTYNYGVTEYDKGNAYAQIAIGTDDVYKTAEVVKLFGGQVVREPGPLPGINTKITSILDPDGWKSVFVDNIDFAKELE

>OsglyI3

MTMTMSNEQGKPEANVRGGRRSGHRVHARHLPGLPTLAAAPARTNGSSWAPGKTEHCTAQHKRRLQAVRDKPQQASVMASEGAVSPAFAYTVVYVKDVAKSAAFYSAAFGYTVRRLDQSHKWAELESGTTTIAFTPLHQRETDALTGAVQLPDSAGERGPVEICFDYADVDAAYRRAVDSGAVPVSPPEQKSWGQKVGYVRDIDGIIVRMGSHVRA

>OsglyI4

MATLQLNHVARETDDVRRLAAFYEEVLGFERVASPNYPAFQVAWLRLPGTPGVALHIIERDPAAAPAAVAPGAAGAPPAQLPRRHHLAFSVADYDGFLTGLKARGTDVFEKTQPDGRTRQVFFFDPDGNGLEVTSSGTGDM

>OsGLYI-5

MGSEAPDPAVAASVPLVRLNHVSFQCTSVEKSVDFYRRVLGFELIKRPESLNFNGAWLYKYGMGIHLLQRGDDADGCSIPTRPLPAINPMGNHVSFQCSDMAVMKARLRAMDREFVVRKVWDGETVVDQLFFHDPDGNMIEVCNCENLPVIPLIVASTPGLPELLPPAMQTNVHG

>OsglyI6

MVNTAAVAAAKGSRGSGLPLASLNHISIVCRSLQESLTFYTDVLGFFPVRRPGSFDFDGAWLFNYGIGIHLLQAEDPDSLPGKTEINPKDNHISFQCESMVAVERRLKELGIPYIQRCVEEGGIYVDQIFFHDPDGFMIEICNCDNLPVVPLGADQPLVMAACKRAAVIKQQQQASSSPATAAAAAQCAVPSSTKAIHVGEEAHISCA

>OsglyI7

MARLLLPLPIAAAAASRLRLPVLSSSVARREALLFGGRVAAARAPVRLARRGVSAGAEAGGSSSAAAAAQVIGQDEAVEWVKKDRRRMLHVVYRVGDLDKTIKFYTECLGMKLLRKRDIPEERYTNAFLGYGPEDSHFVVELTYNYGVESYDIGTAFGHFGIAVEDVAKTVDLIKAKGGTVTREPGPVKGGKSVIAFIEDPDGYKFELIERGPTPEPLCQVMLRVGDLDHAINFYEKAFGMELLRKRDNPQYKYTIAMMGYGPEDKNAVLELTYNYGVKEYDKGNAYAQIAISTDDVYKTAEVIRQNGGQITREPGPLPGINTKITACTDPDGWKTVFVDNVDFLKELEE

>OsglyI8

MAAAAIAAASLLPSSAFALRRLSSAANVSRFAQLKRFDRARRFAPAAAMSTSSGPKEAPANNPGLQAPSEKDPATKGYFMQQTMFRVKDPKVSLDFYSRVMGMSLLKRLDFPEMKFSLYFLGYEDVESAPTDPVKRTVWTFGQRATLELTHNWGTENDPEFKGYHNGNSDPRGFGHIGVTVHDVYKACERFERLGVEFVKKPDDGKMKGIAFIKDPDGYWIEIFDLNRIGAVTAEAS

>OsGLYI-9

MAARCLSSLALLSPSPSSSGKVSAMASPPVPSSAAPRRRPGTRLSVATGGEQLVTAQEASQEPAYGVVSIHHVGILCENLERSMAFYKDLLGLKVNPARPTDKLPYRGAWLWVGSEMIHLMELPNPDPLTGRPEHGGRDRHTCMAIKDVLKLKEIFDKAGIKYTLSKSGRPAIFARDPDGNALEFTQV

>OsglyI10

MAGCRRPTTEMGEVCKRVAPSVREEEEEEENGDGGGVDPAAESSSAKLYEDVPEMPLMALNHISRLCKSIDASVRFYVKALGFVLIHRPPALDFNGAWLFNYGVGIHLVQRDDARRAPDVNPGDLDPMDNHISFQCEDMGMMEKRLNEMGIEYMKRTINEEEGSPIDQLFFKDPDGFMIEICNCENLELVPAGALGRLRLPRDRHNPPLRMAAAGNDEA

>OsglyI11

MASGSEAEKSPEVVLEWPKKDKKRLLHAVYRVGDLDRTIKCYTECFGMKLLRKRDVPEEKYTNAFLGFGPEDTNFALELTYNYGVDKYDIGAGFGHFAIATEDVYKLAEKIKSSCCCKITREPGPVKGGSTVIAFAQDPDGYMFELIQRGPTPEPLCQVMLRVGDLDRSIKFYEKALGMKLLRKKDVPDYKYTIAMLGYADEDKTTVIELTYNYGVTEYTKGNAYAQVAIGTEDVYKSAEAVELVTKELGGKILRQPGPLPGLNTKIASFLDPDGWKVVLVDNADFLKELQ

>ATGLYI-1

MAANMMRPAFAYTVVYVKDVAKSVEFYSRAFGHNVRRLDESHRWGELESGQTTIAFTPLHQHETDDLTGKVQATQSARERAPIEVCFCYPDVDAAFKRAVENGAEAVSKPEDKEWGQKVGYVRDIDGIVVRIGSHVK

>ATGLYI-2

MSSYSIASAISRISPLIRFVKPYSTGFSFITCACNSTRRPKRFDQLCVFSMASEARESPANNPGLSTNRDEATKGYIMQQTMFRIKDPKASLDFYSRVLGMSLLKRLDFSEMKFSLYFLGYEDTTTAPTDPTERTVWTFGQPATIELTHNWGTESDPEFKGYHNGNSEPRGFGHIGVTVDDVHKACERFEELGVEFAKKPNDGKMKNIAFIKDPDGYWIEIFDLKTIGTTTVNAA

>ATGLYI-3

MNEIASASMLRLCQCFISICNVHFVSMRAAESSFLLSRNMAEASDLLEWPKKDNRRFLHVVYRVGDLDRTIEFYTEVFGMKLLRKRDIPEEKYSNAFLGFGPETSNFVVELTYNYGVSSYDIGTGFGHFAISTQDVSKLVENVRAKGGNVTREPGPVKGGGSVIAFVKDPDGYTFELIQRGPTPEPFCQVMLRVGDLDRAIKFYEKALGMRLLRKIERPEYKYTIGMMGYAEEYESIVLELTYNYDVTEYTKGNAYAQIAIGTDDVYKSGEVIKIVNQELGGKITREAGPLPGLGTKIVSFLDPDGWKTVLVDNKDFLKELE

>ATGLYI-4

MKEDAGNPLHLTSLNHVSVLCRSVDESMNFYQKVLGFIPIRRPESLNFEGAWLFGHGIGIHLLCAPEPEKLPKKTAINPKDNHISFQCESMGVVEKKLEEMGIDYVRALVEEGGIQVDQLFFHDPDGFMIEICNCDSLPVVPLVGEMARSCSRVKLHQMVQPQPQTQIHQVVYP

>ATGLYI-5

MATASFRWILQLHRDVPKAARFYEKGLDFSVNVVTLRWAELQSGPLKLALMQAPSEHVMSEKGYSSLLSFTVADINTTISKLMELGAELDGSIKYEVHGKVASVRCLDGHVLGLYEPS

>ATGLYI-6

MVRIIPMAASSIRPSLACFSDSPRFPISLLSRNLSRTLHVPQSQLFGLTSHKLLRRSVNCLGVAESGKAAQATTQDDLLTWVKNDKRRMLHVVYRVGDMDRTIKFYTECLGMKLLRKRDIPEEKYTNAFLGYGPEDSHFVIELTYNYGVDKYDIGAGFGHFGIAVDDVAKTVELVKAKGGKVSREPGPVKGGKTVIAFIEDPDGYKFELLERGPTPEPLCQVMLRVGDLDRAIKFYEKAFGMELLRTRDNPEYKYTIAMMGYGPEDKFPVLELTYNYGVTEYDKGNAYAQIAIGTDDVYKTAEAIKLFGGKITREPGPLPGISTKITACLDPDGWKSVFVDNIDFLKELE

>ATGLYI-7

MKDETGNPLHIKSLNHISLLCRSVEESISFYQNVLGFLPIRRPDSFDFDGAWLFGHGIGIHLLQSPEPEKLLKKTEINPKDNHISFQCESMEAVEKKLKEMEIEYVRAVVEEGGIQVDQLFFHDPDAFMIEICNCDSLPVIPLAGEMARSCSRLNIRQLVQPTQIHP

>ATGLYI-8

MEEKKKKGDDELNSKPPLMALNHVSRLCKDVKKSLEFYTKVLGFVEIERPASFDFDGAWLFNYGVGIHLVQAKDQDKLPSDTDHLDPMDNHISFQCEDMEALEKRLKEVKVKYIKRTVGDEKDAAIDQLFFNDPDGFMVEICNCENLELVPCHSADAIRLPEDRHAPPVALPDSSNRRMPQPNS

>ATGLYI-9

MASLGHIARESSDITRLAQFYKEVFGFEEIESPDFGDLQVVWLNLPGAFAMHIIQRNPSTNLPEGPYSATSAVKDPSHLPMGHHICFSVPNFDSFLHSLKEKGIETFQKSLPDGKVKQVFFFDPDGNGLEVASRS

>ATGLYI-10

MATASFRWILQLHRDVPKAARFYAQGLDFSVNVVTLRWAELHSGPIKLALMQSPSNHVAEKGYSSLLSFTVTDINTTVTKLMALGAELDGTIKYEIHGKVAAMKCPDGYMLGLYEAA

>ATGLYI-11

MASIFRPSSASLDLRPKVICTNLSTKERFEFQKKSVRKERINVRFYSLKAKAQGSSIEGISVVQEKELNNKTDYGVVGVHHVGLLCENLERSLEFYQNILGLEINEARPHDKLPYRGAWLWVGSEMIHLMELPNPDPLTGRPEHGGRDRHACIAIRDVSNLKEILDKAGIAYTMSKSGRPAIFTRDPDANALEFTQV

>GmGLYI-1

MSSSIRPSLSSFMLPSLASCNPSQKLSLFRLGSGIRQFHKFGLKASRFLRHDDKCMRVMAFGNMSTAATQENVLDWVKHDKRRMLHVVYRVGDLDKSIKFYRECLGMKLLRKRDMQEQKYTNAFLGYGPEDAHFVVELTYSNSYGIEKYDIGDGFGHFGIAIDDISRIVELVRAKGGKITREPSPVKGGNTTIAYIEDPDGYQFELLERVPSPEPLCKVMLRVGDLDRSIKFYEKAFGMELLRTQDDPESKSTIGILGYGPEEKNTVLELTYNYGVTNYDKGDAYAQITIDTDDVYKTAEAIKLAGGKITREPGPIPVMKTKITSCVDPDGWKTVFVDNVDFRRELE

>GmGLYI-2

MKMEIEEVGNCEALPLLSLNHVSLLCRSVWVSMRFYEDVLGFVPIKRPSSFKFTGAWFYNYGIGIHLIENPNIDEFDTCVNEERPINPKDNHISFQCTDVELVKKRLEERGMRYVTAVVEEGGIQVDQVFFHDPDGYMIELCNCENIPIIPISSCSFKPRGHSFKKAAPNKCGFMENVMMESLSTDMINFSF

>GmGLYI-3

MSSSLMLPAASMLRPCTTSSSSCTSSRRLALFHLVSTGSIALPQAQLFGAKGPELLRVVEASAAEKLAQPEKDLFDWVKNDNRRFLHVVYRVGDLEKTIKFYTECLGMKLLRQRDIPEDRYSNAFLGYGPEDSNFTVELTYNYGVDNYDIGSGFGHFGVAVEDIYKRVDLVKAKGGKVTREPGPVKDGSAVIAFIEDPDGYKFELLERRPTSEPLCQVMLRVGDLDRAIAFYEKAVGMKLLRKRDNPEQKYTVAFMGYGPEDKNTVLELTYNYGVTNYDKGNGYAQIAIGTNDVYKTAEAIKLCGGKIIREPGPLPGINTKIVACLDPDGWKLAFVDNVDFLKELE

>GmGLYI-4

MVLVRVVPMASSSSIRPTLSSLRFLTPSSLSLSNPSSRISFSHLPSPSVSQSNSFGLKASRELRQHGNSTRIMASGDVSQSISAASPENVLEWVKQDKRRMLHVVYRVGDLDRTIKFYTECLGMKLLRKRDIPEEKYTNAFLGYGPEDSHFVIELTYNYGVDKYDIGTGFGHFGIAVDDVAKAVELIRAKGGKITREPGPVKGGRSVIAFIEDPDGYKFELIERGPTPEPLCQVMLRVGDLNRSIEFYEKAFGMELLRTRDNPEYKYTIAMLGYGPEDKSTVLELTYNYGVTEYDKGNAYAQIAVGTDDVYKTAEAIKLAGGKITREPGALPGINTKITACLDPDGWKSYITRSGRSVFCLLWHDT

>GmGLYI-5

LPQAQLFGAEKIAQPEKNLFDWVKNDNRRFLHVVYRVGDLEKTIKYALLRKRDIPEDRYSNAFLGYGPEESNFTVELTYNYGVDNYDIGSGFGHFGVAGRLITREPGPVKDGSAVIALIEDPDGYKFELLERRPTSEPLCQVMLRVGDIDRAAGMKLLRKRDNPEQKYTVAFMGYGPEYMNSVLELTYNYGVTNYDKGNGYAQIAIGTNDVYKTAEAIKLCGRKIIREPGPLPGINTKIVACLDPDGWKLAFVDNVDFLKELE

>GmGLYI-6

MKESVGNPLRLQSVNHISLICRSVEQSMDFYQNVLGFYPIRRPGSLDFDGAWLFGYGIGIHLLEAENPEKLPKKKEINPKDNHISFQCESMVAVEKKLKEMEIDYVRATVEEGGIQVDQLFFHDPDGFMIEICNCDSLPVIPLVGEVARSCSLVNLEKMQNQQQIQKMLQQL

>GmGLYI-7

MADLLEWSKQDKKRMLHVVYRVGDLDRTIKFYTECLGMKLLRQRDIPEEKYANAFLGFGPEESHFVVELTYNYGVTSYDIGDGFGHFAIATQDIYKLVEHIRAKGGNITREPGPVQGGTTVIAFVKDPDGYTFGLIQRPTVHDPFCQVMLRVGDLERSIKFYEKALGMKVVRKVDKPEYKYTIAMLGYGEEHETTVLELTYNYGVTEYSKGNAYAQIAIGTDDVYKSAEVVNQVIKEVGGKITRQPGPIPGLNTKTTSFLDPDGWKTVLVDNVDFLEELK

>GmGLYI-8

MVLVRLVPMASSSIRPALSTPSSFSLFSPSRRISFSHLPSPSVSQSNSFGLKASRVLRQYGNSTRIMASGDLSHSVAAASPENVLEWVKQDKRRMLHVVYRVGDLDRTIKFYTECLGMKLLRKRDIPEEKYTNAFLGYGPEDSHFVIELTYNYGVDKYDIGTGFGHFGIAVDDVAKAVELIRAKGGKITREPGPVKGGRSVIAFIEDPDGYKFELIERGPTPEPLCQVMLRVGDLNRSIEFYEKAFGMELLRTRDNPEYKYTIAMLGYGPEDKSTVLELTYNYGVTEYDKGNAYAQIAIGTDDVYKTAEAIKLAGGKITREPGPLPGINTKITACLDPDGWKSVFVDNVDFLKELE

>GmGLYI-9

MKESTMGNPLRLQSVNHISLICRSVEQSMDFYQNVLGFYPIRRPGSLDFDGAWLFGYGIGIHLLEAENPENLPKKKEINPKDNHISFQCESMEPVEKKLKEMEIDYVRATVEEGRIQVDQLFFHDPDDFMIEICNCDSLSR

>GmGLYI-10

MLLYKATRPQCRRLFWFVVLCLAIFTIFTEHLHSTLNMAEATQSNAELLEWPKKDKRRFLHVVYRVGDLDRTIKFYTECFGMKLLRKRDIPEEKYANAFLGFGPEQSHFVVELTYNYGVTSYDIGTGFGHFAIATPDVYKLVEDIRAKGGNITREPGPVKGGKSVIAFVKDPDGYAFELIQRSSTPEPLCQVMLRVGDLERSIKFYEKTLGLRVVKKTDRPEYKYTIAMLGYAEEHETTVLELTYNYGVTEYTKGNAYAQVAIGTDDVYKSAEVVNIVTQELGGKITRQPGPVPGLNTKITSFLDPDGWKTVLVDNQDFLKELE

>GmGLYI-11

MASSIRPSLSSFMLPSLRSCNPSEKLSLFHLGSGIRLYHKFGLKSSRLLRHDDNKCMRVMASGNMSTAATQENVLDWVKHDKRRMLHVVYRVGDLDKSIKFYRECLGMKLLRKRDMQEQRYTNAFLGYGPEDAHFVAELTYNYGIDKYDIGDGFGHFGLAVDDISRIVELVRAKGGKITREPSPVKGGNSTIAYIEDPDGYQFELSERVSSPEPLSKVMLRVGDLDRSIKFYEKAFGMELLRTQDDPESKSTIAILGYGPEEKNTVLELTYNYGVTDYDKGDAYAQITIGTDDVYKTAEAIKLAGGKITREPGPVPGIKTKITLCVDPDGWKTVFVDNVDFRRELE

>GmGLYI-12

KEGKGKEENPPPLLAMNHVSRLCRNVKESIDFYTKVLGFVLIERPQALDFEGAWLFNYGVGIHLCEDLEAMEKKLKEKNVKYMKRTLEREDGTTMDQIFFNDPDGFMVEI

>GmGLYI-13

MKMEIEEVGNCEALPLLSLNHVSLLCRSVWESMRFYEDVLGFVPIKRPSSFKFTGAWFYNYGIGIHLIENPNIDEFDTCVVEERPINPKDNHISFQCTDVELVKKRLEERGMRYVTAVVEEGGIQVDQVFFHDPDGYMIELCNCENIPIIPISSCSFKPRGHSFKKAAPNKCGFMENVMMESLSTDMINFSF

>GmGLYI-14

MKDSSSAMVHSMRRSFFNFCLTEKAQLDLYGHSNRINCDCSEPKESPSNNPGLHTTPDQATKAYFTQQTMFRIKDPKVSLDFYSRVLGTYLLKRLDFLEMKFSLYFMGYEDTTKAPSNPVERTVWTFSQKATMELTDNWGTENDPEFKGYHNGNSEPLGYGHIGIAVDDTYKACERFQNLGVEFVTKPDDGFFSQHYCFKHYFQVFFILELFSAGEIKGLAFIKDPDGYWIELFDLKILGGEQAAAHA

>GmGLYI-15

MTVTASLHRLSRLRFIAKPQPFLSPHSIPSHFSLTPKTKKANRFRFLSMAAEPKESPSNNPGLHTTPDEATKGYIMQQTMFRIKDPKVSLDFYSRVLGMSLLKRLDFPEMKFSLYFMGYENTAEAPSNPIDKVVWTFSQKATIELTHNWGTESDPEFKGYHNGNSEPRGFGHIGVTVDDTYKACERFQNLGVEFVKKPEDGKMKGIAFIKDPDGYWIEIFDRKTIGNVTQTAA

>GmGLYI-16

MAATASLHRLSRLRFIAKPQPFLSPHSTPSHFSLTPKTKKPNRFRFRFRSMAAEPKESPSNNPGLHTTPDEATKGYIMQQTMFRIKDPKVSLDFYSRVLGMSLLKRLDFPEMKFSLYFMGYEDTTEAPSNPIDKVVWTFSQKATIELTHNWGTESDPEFKGYHNGNSEPRGFGHIGITVDDTYKACERFQNLGVEFVKKPDDGKMKGIAFIKDPDGYWIEIFDRKTIGNVTQAPA

>GmGLYI-17

MEKMELAETPLPLLSLNHVSFVCKSVSESVKFYEDVLGFLLIKRPSSFKFEGAWLFNYGIGIHLLESEKVPVKKREINPKENHISFQCSDMKVIMQKLDAMKIEYVTAVVEEGGIKVDQLFFHDPDGYMIEICNCQNLPVLPISSCPLKQLGGEATFKINCFAEESMSMLMMDNFVMDMLKISI

>GmGLYI-18

MSCCSCSAMAFLLKAPSFLPPLNQKLNYTHKSFSPINLQSKFYHASVRNGRWNVPSMTIKAQAAVEGDVLLDEESICVNEESDYGVVCMHHVGILCENLERSLDFYQNVLGLKINEARPHNKLPYRGAWLWVGSEMIHLMELPNPDPLTGRPQHGGRDRHTCIAIRDVSKLKAIFDKAGIAYTLSHSGRPAIFTRDPDANALEFTQVDD

>GmGLYI-19

MANPLQLKSLNHISIVCASVEKSVDFYVNVLGFSPIKRPSSLDFNGAWLFNYGIGIHLLQSENPEGMPKTAPINPKDNHISFQCESIAAVEKRLQQMKIEYVKNRVEESGTYVDQLFFHDPDGMMIEICNCDNIPVVPLTEDKVWSCSRFNCNIQNHQQQIQQMIPM

>GmGLYI-20

MKENVGNPLHLKSVNHISLICTSVKESINFYQNLLGFFPIRRPGSFDFDGAWLFGYGIGIHLLQAEDPDNVPRKTKINPKDNHISFQCESMGAVEKKLGEMEIEYVHATVEEGGIKVDQLFFHDPDGFMIEICNCDSLPVIPLAASGNNNGMVRSCSRLNLQILQQIHQFLNQ

>GmGLYI-21

MAEATQSNAELLEWPKKDKRRFLHVVYRVGDLDRTIKFYTECFGMKLLRKRDIPEEKYANAFLGFGPEQSHFVVELTYNYGVTSYDIGTGFGHFAIATPDVYKLVEDIRAKGGNVTREPGPVKGGKSVIAFVKDPDGYAFELIQRPSTPEPLCQVMLRVGDLERSIKFYEKALGLRVVKKTDRPEYKYTIAMLGYAEEHETTVLELTYNYGVTEYTKGNAYAQVAIGTDDVYKSAEVVNIVTQELGGKITRQPGPIPGLNTKITAFLDPDGWKTVLVDNQDFLKELE

>GmGLYI-22

MGKMEPLPLLSLNHVSFVCKSVSESVKFYQDVLGFVLIKRPSSFKFEGAWLFNYGIGIHLLESEKVPVEKREINPKENHISFQCSDMKVIMQKLDAMKIEYVRAVVEEGGIKVDQLFFHDPDGYMIEICNCQNLPVLPISSCPLKQLAAGEATTLNINCFADESVSMLMMDNLVMDMLKISI

>GmGLYI-23

MNCCSAMASLLKSPSFLSPLNQKLNYVSFSPMTTNLQSKFCRASVRNGRWHVPSLTIKSQAAVEGDVLEKESVSINEESDYGVVCMHHVGILCENLERSLEFYQNVLGLKINEARPHDKLPYRGAWLWVGSEMIHLMELPNPDPLTGRAQHGGRDRHTCIAIRDVSKLKAIFDKAGIPYTLSHSGRPAIFARDPDANALEFTQVDG

>GmGLYI-24

MANPLQLKSLNHISIVCASVEKSVDFYVNVLGFSPIKRPSSLDFNGAWLFNYGIGIHLLQSEDPEGMPKLVPINPKDNHISFQHSSGGKRLQQMKIEYVKNRVEENGMMIEICNCDNIPVVPLPEDKVWSCSRFNCNIQNRQQQI
