# Supplementary material for: Genome-wide analysis and expression profiling of glyoxalase gene families in soybean (Glycine max) indicate their development and abiotic stress specific response
Source: BMC Plant Biol. 2016 Apr 16;16:87. doi: 10.1186/s12870-016-0773-9 (PMC4833937; doi:10.1186/s12870-016-0773-9)
Supplement: Additional file 5: — Protein sequences used for phylogenetic analysis of GLYII. (DOCX 17 kb) [file 12870_2016_773_MOESM5_ESM.docx]

>Brassica juncea

MLSKACSLVASSLPRCSSSAAPTIREAAVVAAKRAQENGLGKPLLYGIGTLLVMPLRTLHGVGRMFGAGRFLCNMTSVSSSLQIELVPCLQDNYAYILHDVDTGTVGVVDPSEATPIINALEKRNQNLTYILNTHHHYDHTGGNLELKAKYGAKVIGSAKDRDRIPGIDITLSEGDTWMFAGHQVLVMETPGHTSGHVCYHFPGSGAIFTGDTLFSLSCGKLFEGTPQQMYSSLQKIIALPDETRVYCGHEYTLSNSKFALSIEPGNKDLQEYAANAADLRKRNTPTVPTTIGREKQCNPFLRTSSPEIKNTLSIPDHFDDARVLEVVRRAKDNF

>Pennisetum americanum

MRMLSKACSLVASSLPRCSSSAAPTIRGQPSLLPSVRKEWLDKPLLYGIGTLLVMPLTTLHGVGRMFGAGRFLCNMTSVSSSLQIELVPCLQDNYAYILHDVDTGTVGVVDPSEATPIINALEKRNQNLTYILNTHHHYDHTGGNLELKAKYGAKVIGSAKDRDRIPGIDITLSEGDTWMFAGHQVLVMETPGHTSGHVCYHFPGSGAIFTGDTLFSLSCGKLFEGTPQQMYSSLQKIIALPDETRVYCGHEYTLSNSKFALSIEPGNKDLQEYAANAADLRKRNTPTVPTTIGREKQCNPFLRTSSPEIKNTLSIPDHFDDARVLEVVRRAKDNF

>Leersia perrieri

MRMLSKACSLVASSLPRCSSSAPSIRGQPSLLPSVRKEWLGKPLLYGIGTLLVMPLRTLHGVGRMFGAGRFLCNMTSISSSLQIELVPCLQDNYAYILHDVDTGTVGVVDPSEAMPIINALEKRNQNLTYILNTHHHYDHTGGNLELKAKYGAKVIGSAKDSDRIPGIDITLSEGDTWMFAGHQVLVMETPGHTSGHVSYHFPGSGAIFTGDTLFSLSCGKLFEGTPQQMYSSLQKIVALPDETRVYCGHEYTLSNSKFALSIEPGNKDLQEYAANAADLRKRNTPTVPTTIGREKQCNPFLRTSSPEIKRILSIPDHFDDARVLEVVRRAKDNF

>Sorghum bicolor

MRMLSKACSIVASSLPRCSSSAAPTMRGQPSLLPSVRKQWPGKPLLYGIGTLLVMPLRTLYGVGRVFGAGRFLCNMTSVSSSLQIELVPCLRDNYAYILHDVDTGTVGVVDPSEAMPIINALEKRNQNLTYILNTHHHYDHTGGNLELKAKYGAKVIGSEKDKDRIPGIDITLKEGDTWMFAGHQVLVLETPGHTSGHVCYYFAGSGAIFTGDTLFNLSCGKLFEGTPQQMYSSLQKITALPDDTKVYCGHEYTLSNSKFALSVEPGNKALQEYAANAAELRNKNIPTVPTTIGREKECNPFLRTSNPEIKSTLSIPDHFDEDRVLEVVRRAKDNF

>Hordeum vulgare

MRMFSKACSLVVSSLPRCSSSAPAIRGQPSLIPRVSRKWLGKPLMYGIGTLLVMPLRTLHGVGRMFGAARYLCNMTSVSSSLQIELVPCLQDNYAYILHDVDTGTVGVVDPSEAVPIINALEKRNQNLTYILNTHHHYDHTGGNLELKAKYGAKVIGSEKDKDRIPGIDITLSEGDTWMFAGHQVLVMETPGHTSGHVSFYFPGSGAIFTGDTLFSLSCGKLFEGTPEQMYSSLQKIVALPDVTKVYCGHEYTLSNSRFALSIEPGNEELQEYAASAADLRNKNTPTVPTTIAREKQCNPFLRTSSPEIKKRLSIPDHFDDARVLEVIRRAKDNF

>Triticum aestivum

MRMFSKACSLVVSSLPRCSSSAPAIRGQPSLIPRVSRKWLGKPLMYGIGTLLVMPLRTIHGVGRMFGAARYLCNMTSVSSSLQIELVPCLQDNYAYILHDVDTGTVGVVDPSEAVPIINALEKRNQNLTYILNTHHHYDHTGGNLELKATYGAKVIGSEKDKDRIPGIDITLSEGDTWMFAGHQVLVMETPGHTSGHVSFYFPGSGAIFTGDTLFSLSCGKLFEGTPEQMYSSLQKIVALPDVTKVYCGHEYTLSNSRFALSIEPGNEELQEYAASTADLRNKNTPTVPTTIAREKQCNPFLRTSSPEIKKRLSIPDHFDDARVLEVIRRAKDNF

>Zea mays

MRMLSKACSIVASSLPRCSSSAAAPTMRGQPSLLPSVRKQWPGKPLLYGIGTLLVMPLRTLYGVGRVFGAGRFLCNMTNVSSSLQIELVPCLRDNYAYILHDVDTGTVGVVDPSEAMPIINALEKRNQNLTYILNTHHHYDHTGGNLELKAKYGAKVIGSEKDKDRIPGIDITLKEGDTWMFAGHQVLVLETPGHTTGHVCYYFAGSGAIFTGDTLFNLSCGKLFEGSPQQMYASLQKITALPDDTKVYCGHEYTLSNAKFALSVEPGNKALQEYAANAAELRNKNIPTVPTTIGREKECNPFLRTSNPEIKRTLSVPDHFDEDRVLGVVRRAKDNF

>Setaria italica

MRGQPSLLPSVRKQWLGKPLLYGIGSLLVMPLRTLHGVGRVFGAGRFLCNMTSVSSSLQIELVPCLRDNYAYILHDVDTGTVGVVDPSEAMPIINALEKRNQNLTYILNTHHHYDHTGGNLELKAKYGAKVIGSEKDRDRIPGIDITLKEGDTWMFAGHQVLVLETPGHTSGHVCYYFAGSGAIFTGDTLFNLSCGKLFEGTPQQMYSSLQKIIALPDETKVYCGHEYTLSNSKFALSVEPGNKELQEYAANAAELRNKNIPTVPTTIGREKQCNPFLRTSNPEIKSTLSIPDHFDEDRVLEVVRRAKDNF

>Musa acuminata

MRKQWFGKNLLYGFGTLIVSPIKILRGVNQFLGLPPFFCNITCMSSSLQIELVPCLQDNYAYLLHDIDTGTVGVVDPSEAAPVINVLERRNQNLTYILNTHHHYDHTGGNLELKARYGAKVIGSAKDKDRIPGIDISLHDRETWMFAGHEVLVMETPGHTKGHVSYYFPGCGAVFTGDALFSLSCGKLFEGDPDQMLSSLQQIMSLPDATDVYCGHEYTLSNSKFALSIEPNNQALQEYAAHVAQLRSKMLPTIPTTIKREKQCNPFLRTSSPEIRRKLNIPLSASDAQALGIIRRAKDNF

>Citrus clementina

MQMISRASSAAMASFTCSRGQSGLCVVPGPRQLCLRKGLLYGFMRLLSMPFKTLHLASRSLRVAEFCSISNMSSSLQIELVPCLRDNYAYLLHDMDTGTVGVVDPSEAVPVIDALSRKNRNLTYILNTHHHHDHTGGNLELKARYGAKVIGSGVDKDRIPGIDIVLNDGDKWMFAGHEVHVIDTPGHTRGHISFYFPGSAAVFTGDTLFSLSCGKLFEGTPGQMFSSLQKIMSLPDDTNVYCGHEYTLSNSKFALSIEPNNEALQSYAAHIAQLRSKGVPTIPTTIKMEKSCNPFLRTSSPEIRQSLRIPDTADDSEALGVIRQAKDNF

>Solanum lycopersicum

MRTFSKIAPSAMASFPCSKSRTGVCVWPGMRQLSLRKNLLYGFMQLLSMPFKTVRGVSRSLRVSKLCSITSTSSSLQIELVPCLQDNYAYLLHDVDTGTVGVVDPSEAVPVIDALSRNNRNLTYILNTHHHHDHTGGNMELKARYGAKVIGSGVDSDRIPGIDIALNDGDQWMFAGHEVFVMETPGHTRGHISFYFPRSKAVFTGDTLFSLSCGKLFEGTPEQMLSSLGKITSLPDDTNVYCGHEYTLSNSKFALSIEPGNEELQSYAAHVANLRRKGLPTIPTTLKAEKLCNPFLRTSSTEIRKLLNIPATADDGEALGAIRRAKDNF

>Jatropha curcas

MASLPCSRARSGLCVWPGARQLCFRKGLLYGFMHLLSMPFKTLRGASRTLKVTQFCSVSNMASSLQIELVPCLKDNYAYLLHDVDTGTVGVVDPSEAVPIIDALSRKNRNLTYILNTHHHHDHTGGNEELKARYGAKVIGPGIDRDRIPGIDIVLNDGDKWMFAGHEVLVMETPGHTRGHVSFYFPGSGAIFTGDTLFSLSCGKLFEGTPEQMHSSLRKIMSLPDDTNIYCGHEYTLSNSKFALSIEPNNEALQSYAAQVAHLRGKSLPTIPTTLKIEKACNPFLRASSTEIRQSLNIPATANDAEALGLIRRAKDNF

>Populus trichocarpa

MQMISKASCAMASLPCSRVRSGIRVRPGTRQLSLRKVIVYGFMRLLSTPFKTLRGASRTLKVAQFCSVSNMSSSLQIELVPCLKDNYAYLLHDVDTGTVGVIDPSEAAPVIDALSRKNRNLTYILNTHHHYDHTGGNEELKARYGAKVIGSGVDRDRIPGIDIVLNDGDNWMFGGHEVLVMETPGHTRGHVSFYFPGSGAIFAGDTLFSLSCGKLFEGTPEQMLSSLRKIMSLPDDTNIYCGHEYTLSNSKFALSIDPNNEALQSYAAHVAHLRSKSLPTIPTKLKVEKACNPFLRTSSTAIRHTLNIPATANDSEALGVIRQAKDNF

>Eucalyptus grandis

MKQLCLRKGLPYGLLRFLSIPFKTLWGASRSLKVTQFCTVSSVSSSLQIELVPCLKDNYAYLLHDVDTGTVGVVDPSESRPIIDALSRKNRNLNYILNTHHHYDHTGGNVELKARYGAKVIGSGMDRDRIPGIDIALNGGDKWMFAGHEVLVIETPGHTRGHISFYFPGSGVIFTGDTLFSLSCGKLFEGTPEQMASSLKKITALPDDTNIYCGHEYTLSNSKFALSIEPNNEALKNYAARVAHQRSKGVATIPTTLKMEKMCNPFLRTSSSEIRQLLSIPATADDAEALGIIRQAKDNF

>Spinacia oleracea

MFSKVPHAMASLPCSRVRGGVSLWPGARQLCLRKGLLYGLMRCLSIPFKTLHGAGQSLGVTRLLCNVSCISTALQIELVPCLRDNYAYLIHDENTGTVGVVDPSEAVPVIDALRKKNRNLNYILNTHHHYDHTGGNIELKERYGAKVIGSGRDKERIPGIDIALNDGDRWMFAGHEVHVIETPGHTKGHINFHFSESGAIFTGDTLFSLSCGRLFEGTPEEMLASLKKIMTLPDETSIFCGHEYTLSNAKFALSIEPNNEALQSYVSRVTQLRNKGLPTIPTTLKLEKSCNPFLRTSSTEIRQSLNIPATAGDSEALRVIRQAKDNF

>Medicago truncatula

MLSKASTTAMSAFSSCSRVRTGFSVWPNVRQLCFRKGILYGFMRLFSTPYKTLRGGASRSLRVARFCSVANMSSSLQIELVPCLSDNYAYILHDIDTGTVGVVDPSEATPVIDALSKKNRNLNYILNTHHHHDHTGGNVELKARYGAKVIGSATDKERIPGIDIHLNDGDKWMFAGHEVQVMDTPGHTRGHISFYFAGSGAIFTGDTLFSLSCGKLFEGTPQEMQSSLGKIMSLPDDTSIYCGHEYTLNNTDFALKLEPGNKELRSYAGHVASLRSKGLPTIPTTLKMEKACNPFLRTSNAQIRQLLNIPATADDAEALGIIRQAKDNF

>Gossypium raimondii

MQMLCKASSAVASFPCSRVRSGQCLWPGMRQLCLRKGLVYGFMRFLSTPFKTLRGASRSLRVAEFCSVSNMSSSLQIELVPCLRDNYAYLLHDADTGTVGVVDPSEAVPIIDALSRKNWNLTYILNTHHHHDHTGGNAVLKARYGAKVIGSGIDKDRIPGIDIVLKDGDKWMFAGHEVRVIETPGHTRGHISFYFTGSGAIFTGDTLFSLSCGKLFEGTPEQMHSSLQRIMSLPDGTNVYCGHEYTLSNSKFALSIEPKNEALRSYAAHVAQLRNKGLPTIPTTLKTEKACNPFLRTSSTEIRQALDIPATMNEAEALGVIRRAKDNF

>Phaseolus vulgaris

MHQILSKTSSAMATFPCSRVRSGLCVWPNVRQLCFRKGILYGFMRLFSTPLKTLRGASRTLRVAQFCSVANMSSSLQIELVPCLKDNYAYLLHDVDTGTVGVVDPSEAVPVIDVLSRKNRNLTYILNTHHHHDHTGGNVELKARYGAKVIGSGTDKERIPGIDIHLNDGDKWMFAGHEVRVMDTPGHTRGHISFYFPGSGAIFTGDTLFSLSCGKLFEGSPKEMMSSLKKIMSLPDDTNIYCGHEYTLNNTKFALSIEPENKELQSYAAHVAYLRSKGLPTIPTTLKVEKACNPFLRTSSGAIRQSLNIATTANDAEALAVIRQTKDNF

>Lotus japonicus

MNDMKARRGYCGWPDVRQLSFRKGLLYGFMRLFSLPLKTLRGASRSLRVDRFCGVVYTSSSLQIELVPCLRDNYAYLIYDVNTGTVGVVDPSEAAPIIDVLSEKNLNLTYILNTHHHDDHTGGNADLKERYGAKVIGSGLDKEKIPGIDIHLSDGDKWMFAGHEVHIMATPGHTQGHISFYFPGSEAIFTGDTLFSLSCGKLLEGTPEQMLSSLEKIMLLPDNTSIYCGHEYTLSDSKFALSIEPGNKELKSYAAHIAHLTSRGFPTIPSTLKMEKACNPFLRTSSSEIRQKLNIAVTADNAEALSVIRQAKDKF

>Zostera marina

MFSKLPPSLMMSSIPCYSRARTTTATPTPSLSMGSSRFNWFRKGLIFCFKSLFSTPYKALTSTTSHSLGACRFLCNISSNTSIQIELVPCLEDNYAYLLYDDDTGTVGVVDPSEALPIKNALAKRNMNLTYILNTHHHYDHTGGNLELKEAFGAKVIGAGSDKKRIPGIDIKLYDGDRWMFAGHEVVVMATPGHTKGHISYYFPKSKVIFTGDTLFSLSCGKLFEGSPEQMLCSLGKIATLPGDTKIYCGHEYTESNAKFALSIDPENEQLREYAKLIKQLRRKNLPTVPTTLKREKLCNPFLRTSSTAIRNTLNIPETASDSEALGIIRQAKDRG

>Selaginella moellendorffii

MGSLAFRVALAPPLVGRSSRHARISGARYRLSRCCFSSQSAMASAALEIELIPCLKDNYAYLLRDASSGAIGVVDPSTAQPVIEALERRGLKLTHIINTHHHWDHTGGNADLKKRYGAQIVAPPGDGIPGIDVPLKDGDTWMLGEHAMKVIGTPGHTRGHVSYYFADSRAVFTGDTLFSIGCGRLFEGSAQQMWSSLSKLAALPDETRVFCGHEYTLSNAKFAMTIEPNNPALNSHFEKVKQLRDSGLATIPSSVGEEKKFNPFLRPASREIRRSLNLSDDASDSDVFTAVRKAKDRA

>ATGLX2-4

MQAISKVSSAASFFRCSRKLVSQPCVRPCVRQLHVRKGLVSGVMKLFSSPLRTLRDAGKSVRISRFCSVSNVSSSLQIELVPCLTDNYAYILHDEDTGTVGVVDPSEAVPVMDALQKNSRNLTYILNTHHHYDHTGGNLELKDRYGAKVIGSAADRDRIPGIDVALKDADKWMFAGHEVHIMETPGHTRGHISFYFPGARAIFTGDTLFSLSCGKLFEGTPEQMLASLQRIIALPDDTSVYCGHEYTLSNSKFALSIEPTNEVLQSYAAYVAELRDKKLPTIPTTMKMEKACNPFLRTENTDIRRALGIPETADEAEALGIIRRAKDNFKA

>ATGLX2-3

MVMTHFSRLRQLLLLQPKFLSSQPRPLRSPPPTFLRSVMGSSSSFSSSSSKLLFRQLFENESSTFTYLLADVSHPDKPALLIDPVDKTVDRDLKLIDELGLKLIYAMNTHVHADHVTGTGLLKTKLPGVKSVISKASGSKADLFLEPGDKVSIGDIYLEVRATPGHTAGCVTYVTGEGADQPQPRMAFTGDAVLIRGCGRTDFQEGSSDQLYESVHSQIFTLPKDTLIYPAHDYKGFEVSTVGEEMQHNPRLTKDKETFKTIMSNLNLSYPKMIDVAVPANMVCGLQDVPSQAN

>ATGLX2-5

MQTISKASSATSFFRCSRKLSSQPCVRQLNIRKSLVCRVMKLVSSPLRTLRGAGKSIRVSKFCSVSNVSSLQIELVPCLKDNYAYILHDEDTGTVGVVDPSEAEPIIDSLKRSGRNLTYILNTHHHYDHTGGNLELKDRYGAKVIGSAMDKDRIPGIDMALKDGDKWMFAGHEVHVMDTPGHTKGHISLYFPGSRAIFTGDTMFSLSCGKLFEGTPKQMLASLQKITSLPDDTSIYCGHEYTLSNSKFALSLEPNNEVLQSYAAHVAELRSKKLPTIPTTVKMEKACNPFLRSSNTDIRRALRIPEAADEAEALGIIRKAKDDF

>ATGLX2-1

MPVISKASSTTTNSSIPSCSRIGGQLCVWPGLRQLCLRKSLLYGVMWLLSMPLKTLRGARKTLKITHFCSISNMPSSLKIELVPCSKDNYAYLLHDEDTGTVGVVDPSEAAPVIEALSRKNWNLTYILNTHHHDDHIGGNAELKERYGAKVIGSAVDKDRIPGIDILLKDSDKWMFAGHEVRILDTPGHTQGHISFYFPGSATIFTGDLIYSLSCGTLSEGTPEQMLSSLQKIVSLPDDTNIYCGRENTAGNLKFALSVEPKNETLQSYATRVAHLRSQGLPSIPTTVKVEKACNPFLRISSKDIRKSLSIPDSATEAEALRRIQRARDRF

>ATGLX2-2

MKIFHVPCLQDNYSYLIIDESTGDAAVVDPVDPEKVIASAEKHQAKIKFVLTTHHHWDHAGGNEKIKQLVPDIKVYGGSLDKVKGCTDAVDNGDKLTLGQDINILALHTPCHTKGHISYYVNGKEGENPAVFTGDTLFVAGCGKFFEGTAEQMYQSLCVTLAALPKPTQVYCGHEYTVKNLEFALTVEPNNGKIQQKLAWARQQRQADLPTIPSTLEEELETNPFMRVDKPEIQEKLGCKSPIDTMREVRNKKDQWRG

>OsGLYII-1

MVALLRSCRRLIPHLSACAAAAPSSSSSCAPRARPISRGLRLLPVVLAMAGYSSGSAAEGRRLLFRQLFEKESSTYTYLLADVGDPEKPAVLIDPVDRTVDRDLNLIKELGLKLVYAMNTHVHADHVTGTGLIKTKLPGVKSVIAKVSKAKADHFIEHGDKIYFGNLFLEVRSTPGHTAGCVTYVTGEGDDQPSPRMAFTGDALLIRACGRTDFQGGSSDELYESVHSQIFTLPKDTLLYPGHDYKGFTVSTVEEEVAYNARLTKDKETFKKIMDNLNLAYPKMIDVAVPANLLCGIQDPPPSKV

>OsGLYII-2

MKIIPVACLEDNYAYLIVDESTKSAAAVDPVEPEKVLAAAAEVGVRIDCVLTTHHHWDHAGGNEKMAQSVPGIKVYGGSLDNVKGCTDQVENGTKLSLGKDIEILCLHTPCHTKGHISYYVTSKEEEDPAVFTGDTLFIAGCGRFFEGTAEQMYQSLCVTLGSLPKPTQVYCGHEYTVKNLKFILTVEPDNEKVKQKLEWAQKQREANQPTIPSTIGEEFETNTFMRVDLPEIQAKFGAKSPVEALREVRKTKDNWKS

> OsGLYII-3

MRMLSKACSLVASSLPRCSSSAAPTIRGQPSLLPSVRKEWLGKPLLYGIGTLLVMPLRTLHGVGRMFGAGRFLCNMTSVSSSLQIELVPCLQDNYAYILHDVDTGTVGVVDPSEATPIINALEKRNQNLTYILNTHHHYDHTGGNLELKAKYGAKVIGSAKDRDRIPGIDITLSEGDTWMFAGHQVLVMETPGHTSGHVCYHFPGSGAIFTGDTLFSLSCGKLFEGTPQQMYSSLQKIIALPDETRVYCGHEYTLSNSKFALSIEPGNKDLQEYAANAADLRKRNTPTVPTTIGREKQCNPFLRTSSPEIKNTLSIPDHFDDARVLEVVRRAKDNF

>GmGLYII-1

RKDENPSHTNYSYLYVVVFVTNLQDNVKGCTDKVENGDKVSLGPDVTVLALLTPCHTQGHISYYVTGKEDEQPAVFTGDTLFIASCGKFFEETAEQMYQSLNVTLASLPKSTRVYRGHEYSVNNLQFAVTLEPDNLRIQKKLAWARNQWQAGQATIPSTIEDELETNPFMRVDLPEIQERVGCKSPVKALGEIRKQKDNWRG

>GmGLYII-2

MKIYHVPCLRDNYSYLIVDKSTKEGAVVDPVEPQKVLEAANSHWVNLKLVLTTHHHGDHAGGNEKIKQLVPGIKVYGSLIDNVIGCTDKVENGDKESLGADIYILCLHTPCHTKGHISYYVTGKEEEQPAVFTGDTLFIADCGKFFKGTAEQMYQSLCVTLGSLPKPTRVYCGHGEKVGCKSPVEALRELRKLKDNWKG

>GmGLYII-3

IVDESTKEGAVVDPVEPQKVLEAANSHGVNNLKLVLTTHHHGDHAGGNEKIKQLVLGMKVYGGSMDNIKGCTDKVENGDKMSLGADINILCLHTPCHTKGHISYCVTGKEEEVLRKEYNKLILKAMPKFIAGCGKFFEGTAEQIYQSLCVTLGSLPKPTRVYCGHEYAVRNLLFALTIEPDNLRIQQKLTWAKNQQQAGQSTIPSTIEEEMETNPFMRVNLPEIQGASLPVEALRELRKLKDKWKGVMELTNYCILHV

>GmGLYII-4

MGDTKERSGFCVWPDARQLCLGKGLLYGFMRLFSIPLKTLRGASRSLRVNQFCSVVNLSSSLQIELVPCLRDNYAYLLHDVDTGTVGVVDPSEAAPIIDALSKKDLNLTYIMNTNHHPDHTGGNAELKERYGAKVIGSEIDKERIPGIDIYLSDGDNWMFAGHEVHILATPGHTEGHVSFYFPGSGAIFTGDTLFSLSCGKLLEGTPKQMLSSLKRIMSLPDDTSIYCGHEYTSSNSKFALSIEPENKELQSYAAHVANLRNKGLPTIPTTVKVEKACNPFLRTWSMEIRQKLNIATTADDAEALGVIQQAKDNF

>GmGLYII-5

MNVLVFERSGFCVWPDARQLCLRKGLLYGFMRLFSIPLKTLRGASRSLRVDQFCSVVNLSSSLQIELVPCLRDNYAYLLHDVDTGTVGVVDPSEAAPIIDALSKKDLNLTYILNTNHHPDHTGGNAELKERYGAKVIGSEIDKERIPGIDIYLSDGDNWMFAGHEVHILATPGHTEGHVSFYFPGSGAIFTGDTLFSLSCGKLLEGTPEQMLSSLKRIMSLPDDTSIYCGHEYTLNNSKFALSIEPENKELQSYATHVSNLRNKGLPTIPTTLKVEKACNPFLRTWSIEIRQKLNIAATADDAEALGVIRQAKDNF

>GmGLYII-6

MPIATKLYASNVTSTLNSKTNSNNGDLIYVILIVIPVIKLFLYSTIHNQNPTTSTLQSLSSIQPFWWQLLFMLQKMLRLHFTTALSHFASKASPFPLTPVSVTVSRAIVCNNPTRFRSQMGSFSTSSSSSSKLLFRQLFEKESSTYTYLLADASHPEKPALLIDPVDRTVDRDLSIIEQLGLKLVYAMNTHVHADHVTGTGLIKSKVPSVKSVISKASGATADLYVEPGDKVQIGDLFLEVRATPGHTKGCVTYVTGDAPDQPQPRMAFTGDTLLIRGCGRTDFQGGSSEQLYKSIHSQILTLPKSTLIYPAHDYKGFTVSTVGEELQNNPRITKDEETFKNIMGNLNLSYPKMIDIAVPANMVCGIQSNPKQAEAS

>GmGLYII-7

MLHMFSKASSAMATFPCSRVKSGLCVWPDVRQLCFRKGMLYGFMRLFSTPLKTLRGASRSLRVTQFCSVANMSSSLQIELVPCLKDNYAYLLHDVDTGTVGVVDPSEAVPIIDALSRKNRNLTYILNTHHHHDHTGGNVELKARYGAKVIGSGTDKERIPGIDIHLNDGDKWMFAGHEVRVMDTPGHTRGHISFYFPGSGAIFTGDTLFSLSCGKLFEGTPQQMLSSLKKIMSLSDDTNIYCGHEYTLNNIKFALSIEPENEELQSYAAQVAYLRSKGLPTIPTTLKVEKACNPFLRTSSAAIRQSLKIAATANDAEALGVIRQAKDNF

>GmGLYII-8

MRIHHIACLQDNYSYLIVDESTKEAAAVDPVEPEKVLEVASSHGLTLKFVLTTHHHWDHAGGNDKIKQLVPGIKVYGGSIENVKGCTDKVENGDKVSLGAEITILALHTPCHTQGHISYYVTGKEDEQPAVFTGDTLFIASCGKFFEGTAEQMYQSLNVTLASLPKSTRVYCGHEYSVNNLQFALTLEPDNLRIQQKLTWARNQRQAGQATIPSTIEDELETNPFMRVDLPEIQERVGCKSPVEALGEIRKQKDNWRG

>GmGLYII-9

MLSKPSSAMPTFPSSMVRSGLCVWPNVRQLCFRKGILYGFMRLFSTPLKTLRGASRSLRVAQFCSVANMSSSLQIELVPCLKDNYAYLLHDVDTGTVGVVDPSEAVPVIDALSRKNRNLTYILNTHHHHDHTGGNVELKARYGAKVIGSGTDKKRIPGIDIHLNDGDKWMFAGHEVRVMDTPGHTQGHISFYFPGSGAIFTGDTLFSLSCGKLFEGTPQQMLSSLKKIMSLPDNTNIYCGHEYTLNNTKFALSIEPENEELQSYAAQVAYLRSKGLPTIPTTLKMEKACNPFLRTSSAAIRQSLNIAATANDAEALGGIRQAKDNF

>GmGLYII-10

LRSQMCSFSTTSFSSSSSKLLFHQLFEKKSSTYTYLLADASHPEKPTLLIDPVDRTVDRDLSLIEQLGLKIVYTMNTHVHADHVTGTGLIKGKVPSVKSVISKASGATVDLYVEPGDKVHIGDLFLEVRATPGHTKGCVTYVTGDAPDQPQPRMAFTGDTLLIRGCGRTGFQIYTCSKLLEQRRKWVKV

>GmGLYII-11

TGKKRIPAIDIHLNDGDKWMCAGHEVRVMDTPGHTQGHISFYFPGSGVIFTGDTFFNLSCGKLFEGTPQQVVLNCTCPFFLFFFF

>GmGLYII-12

MATHRLALIIQNPSNDDEFLLVKQSRPPKFHDEEYDSFVDSDLWDLPSAQLNPLLAESEPPVELELAVSHSESQDVDLRKFDIRSALNEVFGQLGFGAVDGGGWKFHKYVKEAAFGPDLPVNTVFIVGKLVAAEDKDFRDSYRWKSVRSCLNWILEVKPHGDRVGPLVVIGLINESSISTKWKVPPAINYQEYPPGNIIIPMGSRTLRPFHTTNLVVFAPENVSNDSGENNFIVRGDALIVDPGCLSEFYGELEKIVTALPRRLVVFVTHHHPDHVDGLSVIQKCNPDATLLAHEKTMHRISRDVWSLGYTPVTGDEDIDIGGQRLRVIFAPGHTDGHMALLHANTHSLIVGDHCVGQGSATLDIKAGGNMSEYFQTTYKFLELSPHALIPMHGRVNVWPKQMLCGYLKNRRSREANIVKAIEGGAKSLFDIIVYVYSDVDRRAWIAASSNVRLHVDHLAQQHKLPKDFSIQKFKNTCGLHFLSRWIWAYGSGSLSHQIGKSPFLVAGVLAGIAGIAVLYCQRKFTK
